# The hypothalamic RFamide, QRFP, increases feeding and locomotor activity: The role of Gpr103 and orexin receptors

**DOI:** 10.1371/journal.pone.0275604

**Published:** 2022-10-17

**Authors:** Chris Cook, Nicolas Nunn, Amy A. Worth, David A. Bechtold, Todd Suter, Susan Gackeheimer, Lisa Foltz, Paul J. Emmerson, Michael A. Statnick, Simon M. Luckman

**Affiliations:** 1 Division of Diabetes, Endocrinology and Gastroenterology, School of Medicine, University of Manchester, Manchester, United Kingdom; 2 Lilly Research Laboratories, Eli Lilly and Company, Indianapolis, IN, United States of America; 3 Recursion Pharmaceuticals, Salt Lake City, UT, United States of America; Universidade do Estado do Rio de Janeiro, BRAZIL

## Abstract

Here we show that central administration of pyroglutamylated arginine-phenylamine-amide peptide (QRFP/26RFa) increases both food intake and locomotor activity, without any significant effect on energy expenditure, thermogenesis or reward. Germline knock out of either of the mouse QRFP receptor orthologs, *Gpr103a* and *Gpr103b*, did not produce a metabolic phenotype. However, both receptors are required for the effect of centrally administered QRFP to increase feeding and locomotor activity. As central injection of QRFP activated orexin/hypocretin neurons in the lateral hypothalamus, we compared the action of QRFP and orexin on behaviour. Both peptides increased arousal and locomotor activity. However, while orexin increased consummatory behaviour, QRFP also affected other appetitive behaviours. Furthermore, the feeding but not the locomotor response to QRFP, was blocked by co-administration of an orexin receptor 1 antagonist. These results suggest that QRFP agonism induces both appetitive and consummatory behaviour, but only the latter is dependent on orexin/hypocretin receptor signalling.

## Introduction

Deorphanisation of the receptor GPR103 (also known as AQ27 and SP9155) led to the description of a novel, 43-amino acid RFamide, pyroglutamylated arginine-phenylamine-amide peptide (QRFP), expressed in multiple mouse tissues including the brain, eye, kidney, adrenal, heart, thyroid and gonads [1,2]. At the same time, a shorter product of the *Qrfp* gene, 26RFa, was isolated from frog, and shown to increase food intake when injected centrally in mice [3]. The expression of the *Qrfp* mRNA in the rodent brain is restricted to the mediobasal hypothalamic region, surrounding, but not including the ventromedial and arcuate nuclei [3–5]. Since these discoveries, acute metabolic effects have been described for either central or systemic QRFP or 26RFa [6]. However, further indications as to the role for this brain peptidergic system remain obscure. Central dosing of rodents with QRFP/26RFa is generally agreed to have a short-lasting orexigenic effect, which may be mediated by the activation of neuropeptide Y (NPY) neurons in the hypothalamic arcuate nucleus [3–5,7–9]. Takayasu and colleagues also recorded an acute increase in locomotor activity and oxygen consumption [4]. By comparison, medium-term, central infusion with QRFP/26RFa caused increases in body weight and adipose tissue mass, through hyperphagia and potentially reduced thermogenesis, though energy expenditure was not measured directly in this study [7]. Knock-out studies have provided mixed results too. One *Qrfp* knock-out model has a modest lean phenotype, with a small decrease in cumulative food intake and no effect on energy expenditure [10]. A second null mutant has no energy intake or energy expenditure phenotype, though a slight increase in respiratory exchange ratio [11]. *Qrfp* knock-out mice also exhibit reduced risk avoidance and decreased wakefulness in the early dark phase [10]. In a more recent paper, describing chemogenetic stimulation of “Q neurons”, activation of a mixed population of neurons in the hypothalamus and preoptic regions, which included but which was not exclusive to QRFP neurons, increased feeding and core-body temperature [12]. However, the latter effect appears to be dependent on the role of an unidentified population of non-QRFP neurons in the preoptic region.

*Gpr103* mRNA is expressed at high levels in brain (including the retina) of all species studied, whereas its description in other tissues is variable [1,2]. Within the rat brain iodinated 26RFa binding sites are much more widespread than the distribution of *Gpr103* mRNA described by *in situ* hybridisation histology [13]. There may be several reasons for this discrepancy. Firstly, unlike in the human, rodents have two orthologs of the receptor, which are termed Gpr103a and Gpr103b [4,5], which overlap in some, but not all brain regions. A *Gpr103* knock-out mouse model was shown to have normal body weight [14]. However, at the time of publication, it was unknown that two orthologs existed. Secondly, binding to Gpr103 receptors may be in the terminals of neurons and, therefore, remote from the respective mRNA in cell bodies. Thirdly, QRFP has a moderate affinity for the receptors of another RFamide, neuropeptide FF receptor 2 (NPFFR2) [15,16] and so some binding may have been to this receptor.

Due to similarities between the literature on QRFP and orexin/hypocretin [17,18], we hypothesise that their signalling within the brain may be linked. In the present study, we demonstrate central administration of QRFP increases both food intake and locomotor activity, without any significant effect on either energy expenditure or thermogenesis. Germline knock out of either of the cognate mouse QRFP receptors, Gpr103a and Gpr103b, which evolved through a gene duplication event, does not produce a metabolic phenotype. However, both receptors are required for the effect of centrally administered QRFP to increase feeding and locomotor activity. We compare the action of centrally administered QRFP with the behavioural effects of orexin and find that the feeding, but not the locomotor response to QRFP, is blocked by co-administration of an orexin receptor antagonist.

## Results

### Acute actions of central QRFP administration

**Fig 1:**
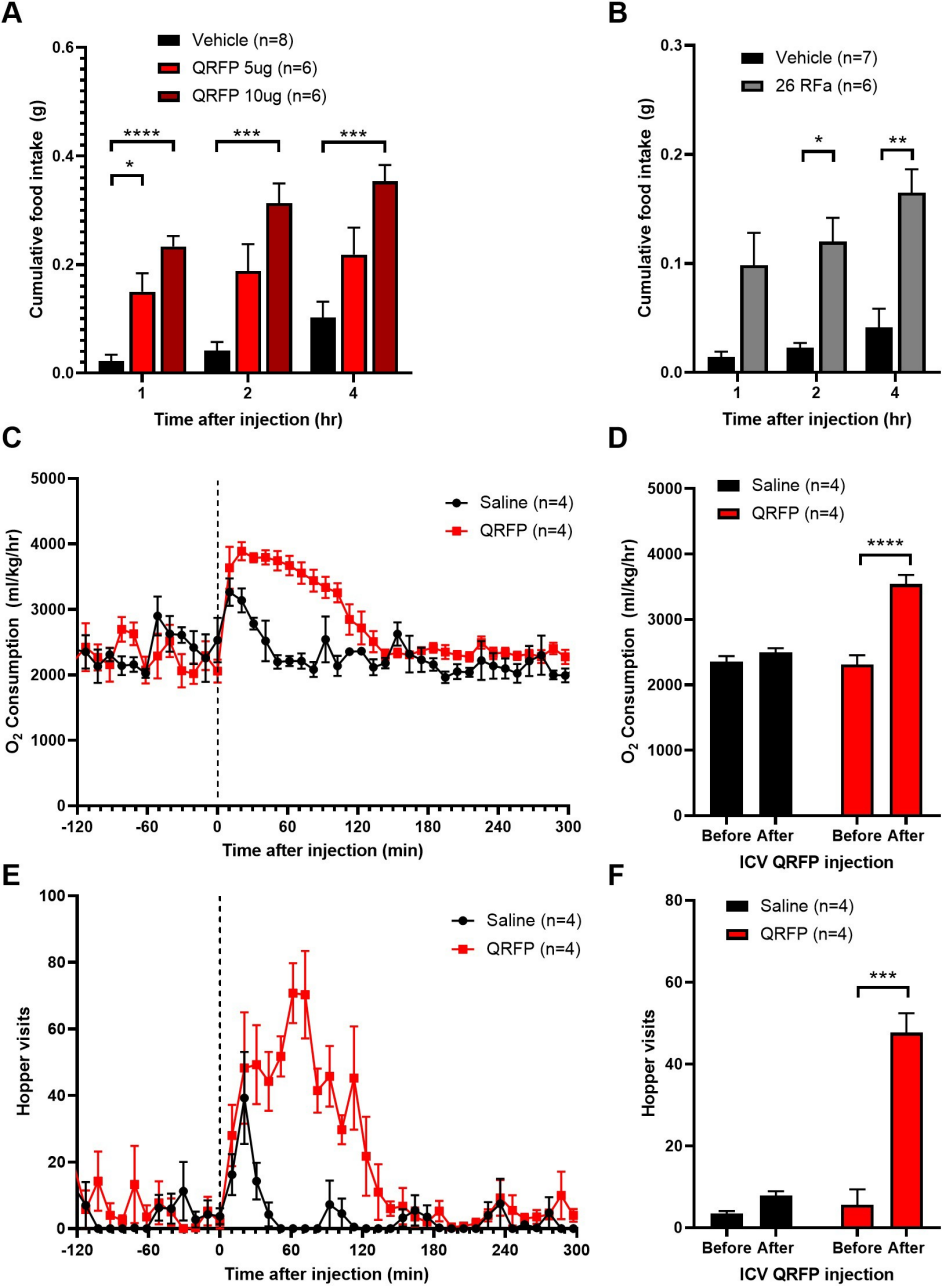
QRFP increases food intake and, acutely, oxygen consumption. Central injection of (A) QRFP (5 and 10 μg, ICV) or (B) 26RFa (5 μg, ICV), the N-terminally truncated form of the peptide, during early daylight causes an acute increase in food intake. The majority of feeding occurs within 2 hours of injection. Repeated measures, two-way ANOVA with Dunnett’s *post hoc* test (QRFP F_2,17_ = 19.46; 26RFa F_1,11_ = 26.23). (C and D) QRFP (10 μg, ICV) increased oxygen consumption (VO_2_) in the two hours after injection in outbred CD1 mice (F_1,6_ = 12.94). (E and F) Food hopper visits increased in the same mice immediately following injection with QRFP (F_1,6_ = 57.9). Two-way ANOVA with Sidak’s *post hoc* test. *p<0.05, **p<0.01, ***p<0.001, ****p<0.0001.

Here we confirmed that both the 43-amino acid QRFP and the shorter peptide, 26RFa, increase food intake when injected centrally (Fig 1A and 1B). It is widely acknowledged that, at least in heterologous cell systems *in vitro*, there is some overlap in affinities for different members of the RFamide receptor family. For example, both QRFP and prolactin-releasing peptide (PrRP) can bind with the neuropeptide FF receptor, NPFFR2. However, QRFP clearly functions as an orexigen, compared with PrRP and NPFF, which are anorexic (S1 Fig), thus it is unlikely that QRFP is having a major effect through PrRP or NPFF receptors *in vivo*. In an early study, long-term treatment with QRFP was reported to increase body weight and adiposity, through hyperphagia and potentially reduced thermogenesis (though energy expenditure was not measured directly) [7]. In our hands, there was an acute increase in oxygen consumption immediately after administration of QRFP (Fig 1C and 1D). However, the increase in oxygen consumption corresponded closely with visits to the food hopper (Fig 1E and 1F) and, thus, is likely to be a simple reflection of increased activity. Moreover, neither QRFP nor 26RFa affected brown adipose tissue temperature, as measured by infrared thermography of the interscapular region (S2 Fig). To test operant responding, mice were trained to press levers to obtain strawberry milkshake rewards through fixed-, then progressive-ratio training. Mice were injected with sub-maximal orexigenic doses of either QRFP or orexin (5 μg and 7 μg ICV, respectively), and a sub-threshold dose of QRFP (2 μg ICV) and tested for operant responding before being returned to their home cage and chow intake measured. As a positive control, orexin increased both breakpoint and correct lever pressing in a progressive-ratio test (S3 Fig, as previously published [19]. In the same mice, neither dose of QRFP affected either breakpoint or lever pressing (S3 Fig), even though the higher dose did cause increased food intake when the mice were returned to their home cage. This suggests that QRFP does not increase food intake through acting on reward pathways.

**Fig 2:**
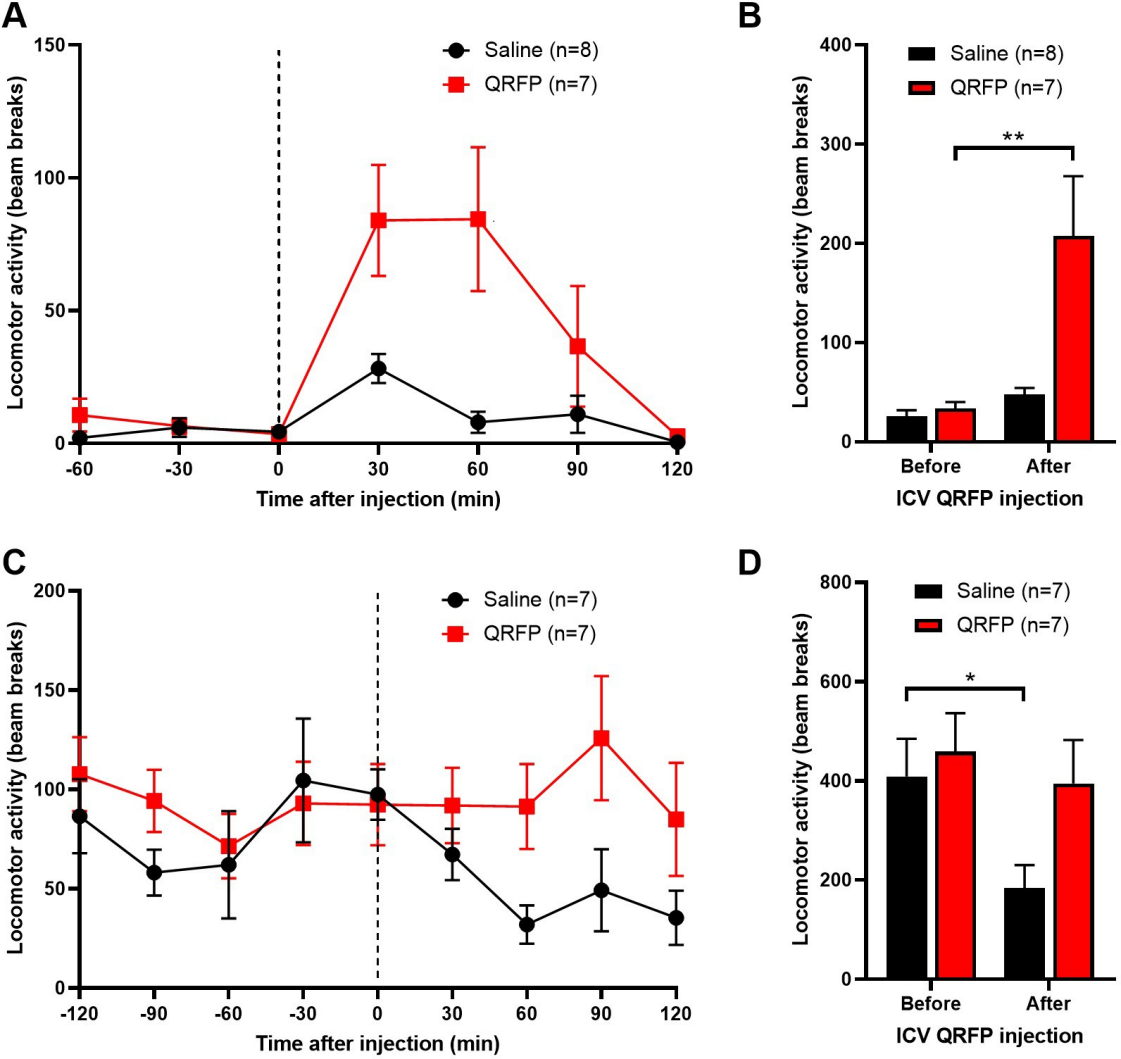
QRFP increases locomotor activity in sated mice. (A and B) Central QRFP (7.5 μg, ICV) induces locomotor activity during the daytime (injections made approximately 3 hours after lights on). Activity is compared in the two-hour periods before and after injection (F_1,13_ = 8.82). (C and D) When given two hours after lights out, QRFP maintains the normal hyperactivity seen in mice after the transition to nighttime (F_1,12_ = 2.18). Two-way ANOVA with Sidak’s *post hoc* test. *p<0.05, **p<0.01.

Since mice are nocturnal, laboratory animals tend to be quiescent during the day. They exhibit high levels of activity in the first couple of hours after lights out, and then settle down to intermittent periods of activity through the night. Firstly, mice were injected ICV with an orexigenic dose of QRFP (7.5 μg ICV) during the daytime, and locomotor activity recorded for the next two hours. QRFP significantly increased the number of beam breaks in the first hour following injection (Fig 2A and 2B). Secondly, mice were injected with the same dose of QRFP two hours after lights out. In accordance with the aforementioned circadian profile, all mice exhibited high levels of activity in the two hours between lights out and the night-time injections. Saline-treated mice then reduced their activity for the subsequent two hours, whereas QRFP-treated mice maintained the high level of activity (Fig 2C and 2D). These mice also showed increased food intake during this period.

### Gpr103a and Gpr103b receptor knock out in mice

Homology searches identified a duplication in the QRFP receptor gene, *Gpr103*, in the rodent superfamily, Muroidea, producing an ortholog (*Gpr103a*) and near ortholog (*Gpr103b*) in mouse [4], rat [5] and hamster (S1 Table). A comparison of nearest neighbour genes suggested no duplication event in other rodent suborders (squirrel, guinea pig) or in lagomorphs (rabbit) (see also [20]). Likewise, homology and nearest neighbour genes analysis identified a single *GPR103* homolog in humans, monkeys, pigs and dogs. Human, whole-body tissue analysis by real-time PCR revealed strong expression of the *GPR103* gene in the eye and reproductive tissues, with lower expression in the brain (S4A Fig). In the mouse, *Gpr103a* expression is high in the eye, spinal cord and brain. *Gpr103b* expression exists in adipose and reproductive tissues but is notably lower in the brain (S4B Fig).

We sourced distinct *Gpr103a* and *Gpr103b* knock-out mice, which we validated by end-point PCR on a panel of metabolic tissues from the two strains (S5 Fig) and by the gross distribution of QRFP receptors in the mouse brain using ^125^I-QRFP binding and autoradiography (S6 Fig). According to the literature, within the rodent brain, *Gpr103a* and *Gpr103b* overlap in some regions, but not others [5,13]. Our results demonstrated that binding to both receptors is found in the lateral septum, nucleus accumbens (core and shell), reuniens thalamic nucleus, zona incerta, nuclei of the hypothalamus, central grey and spinal trigeminal tract. Gpr103a binding is exclusive to the parietal cortex, portions of the amygdala and the brainstem nucleus of the tractus solitarius. Gpr103b binding is exclusive to the caudate putamen and the substantia nigra (pars compacta).

**Fig 3:**
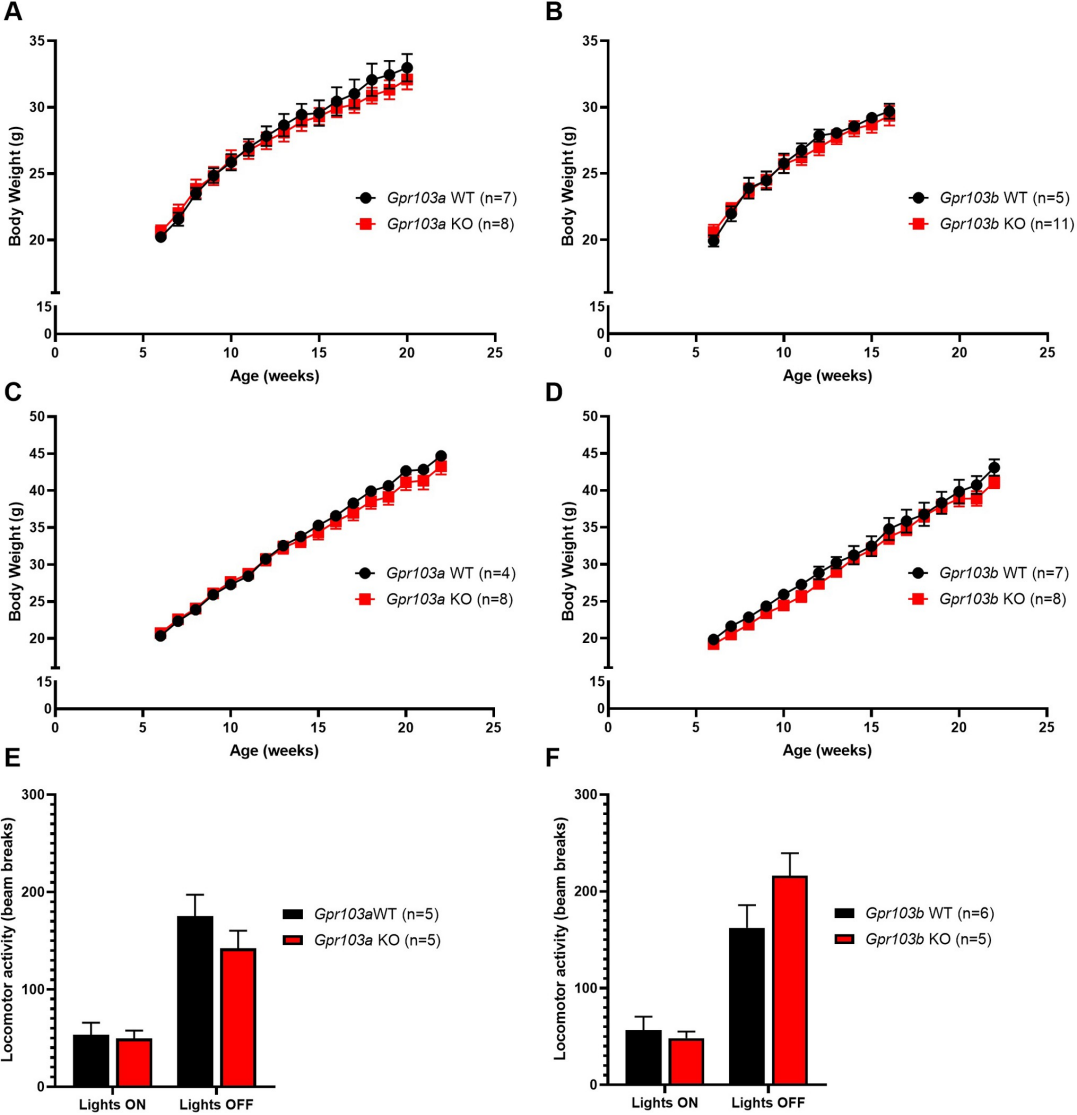
Germline deletion of *Gpr103a* or *Gpr103b* does not produce a metabolic phenotype. Growth curves for male *Gpr103a* knock-out mice and wild-type littermates on (A) normal chow or (B) high-energy diet. (C) Locomotor activity (beam crosses) during the light and dark phases in *Gpr103a* knock-out mice and wild-type littermates. (D and E) Growth curves for *Gpr103b* knock-out mice and wild-type littermates on normal chow or high-energy diet. (F) Locomotor activity in *Gpr103b* knock-out mice.

To determine the metabolic phenotype in receptor null mice, *Gpr103a* and *Gpr103b* knock-out homozygotes and their wild-type littermates were maintained on either normal chow or high-energy diet (HED) from 4 weeks of age. No body-weight phenotype was observed, when monitored until 20 weeks of age, on either diet (Fig 3A–3D). Spontaneous 24-hour and fast-induced re-feeding was the same in both receptor knock-out mice (S2 Table). No obvious differences in food intake or glucose handling were noted (S2 Table). Baribault and co-workers reported expression of *Gpr103* in osteoblasts and that their *Gpr103* knock-out mouse had reduced bone formation [14]. This is interesting as, reportedly, there are gene polymorphisms in a model osteopenic mouse [21]. We found no evidence for abnormal bone development. Furthermore, locomotor activity was monitored during both daytime and night-time phases, but no differences were seen between either *Gpr103a* and *Gpr103b* knock-out mice and their wild-type littermates (Fig 3E and 3F).

**Fig 4:**
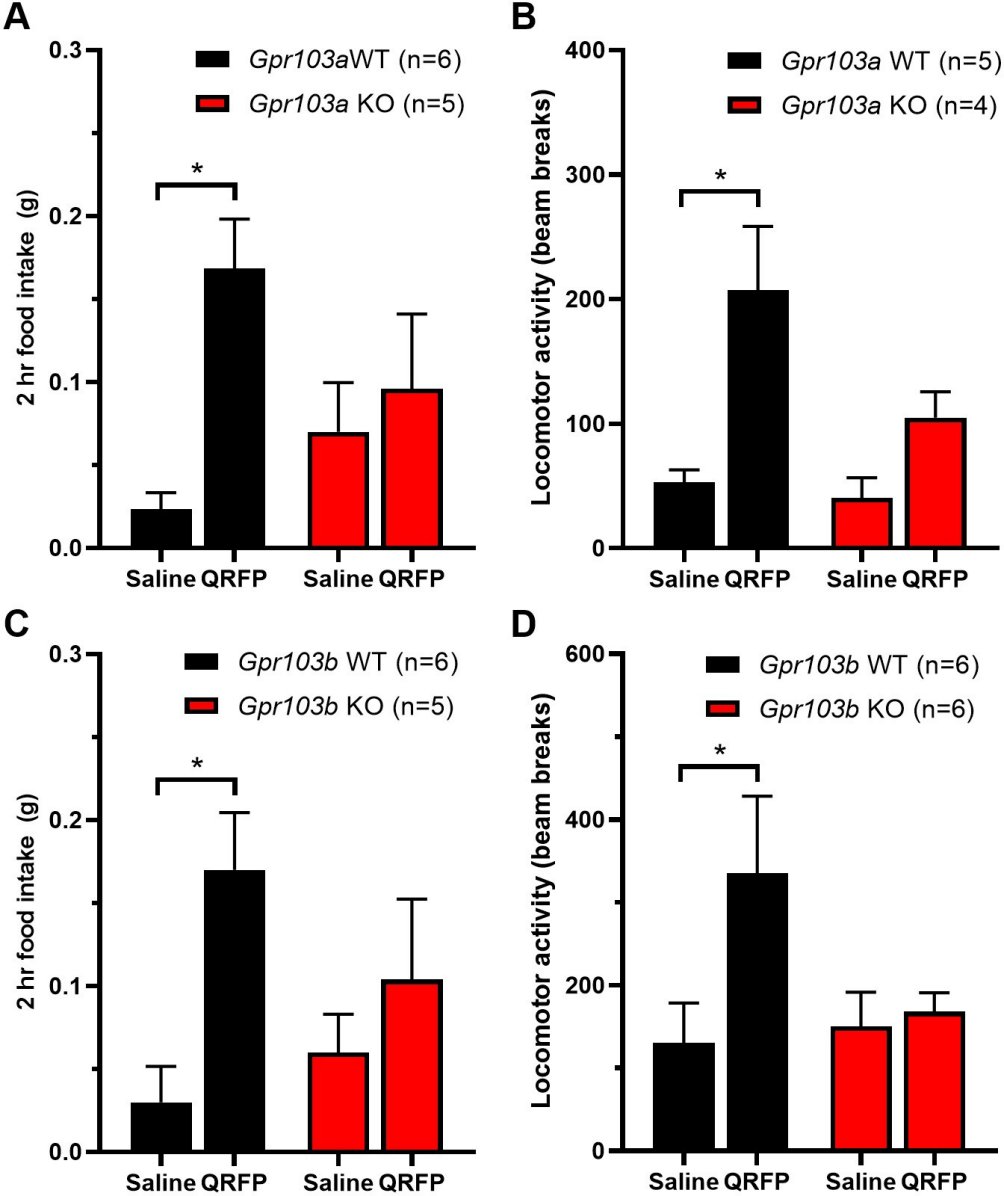
Both *Gpr103a* and *Gpr103b* are required for the acute actions of QRFP. (A and B) ICV QRFP increases food intake and locomotor activity in wild-type but not in *Gpr103a* knock-out littermates (food intake F_1,9_ = 5.52; activity F_1,7_ = 14.2). (C and D) The same is true in *Gpr103b* knock-out mice (food intake F_1,9_ = 6.01; activity F_1,10_ = 11.17). Two-way ANOVA with Sidak’s *post hoc* test. *p<0.05.

As expected, ICV QRFP causes an increase in food intake (Fig 4A and 4B) and locomotor activity in wild-type littermates from both receptor strains (Fig 4C and 4D). However, QRFP did not increase food intake or activity in knock-out littermates of either the *Gpr103a* or *Gpr103b* lines.

### Interaction between QRFP and orexin signalling

**Fig 5:**
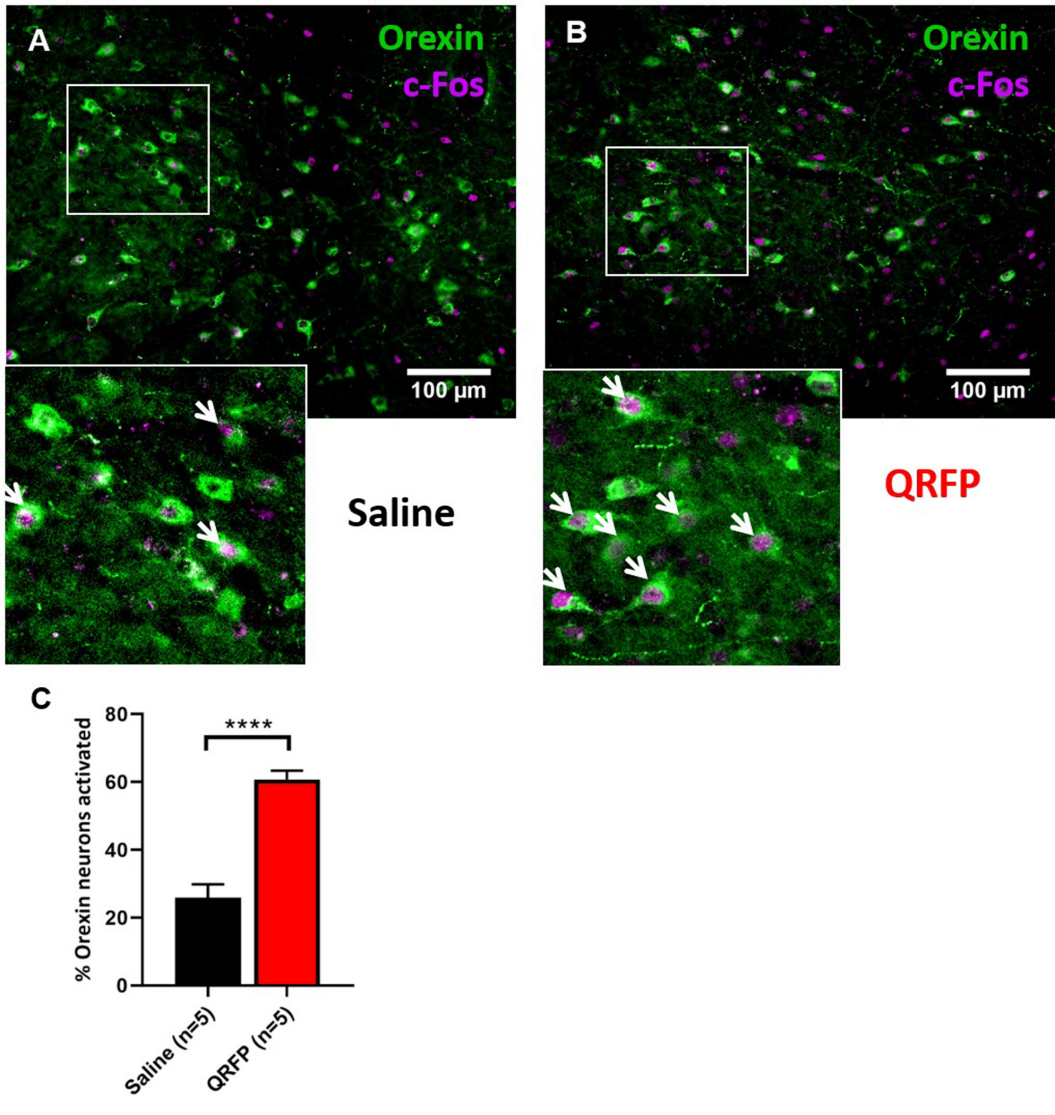
ICV QRFP caused an activation of orexin/hypocretin neurons. Dual-label immunohistochemistry for FOS (magenta) and orexin/hypocretin (green) in the lateral hypothalamus after ICV injection of either (A) saline or (B) QRFP. (C) Orexin/hypocretin neurons are activated by QRFP (t_8_ = 7.36). Student’s t-Test. ****p<0.0001. 3–4 sections per mouse were analysed at approximately -1.8 mm relative to bregma.

In a preliminary experiment, we looked at brain activity patterns following central injection of QRFP or control saline, using immunohistochemistry for the cellular marker, FOS protein. Since the increased food intake after central dosing of rodents with QRFP/26RFa is reported to be reduced by co-administration NPY receptor antagonists [4,9], we carried out this experiment in *Npy*-Cre::eYFP reporter mice. Following lateral ventricular injection of QRFP, relatively little FOS expression was observed within the forebrain, and no significant increase in FOS was observed within any anatomically defined hypothalamic nucleus; namely the paraventricular, suprachiasmatic, ventromedial, dorsomedial or arcuate nuclei (S7 Fig). QRFP did, however, induce a significant increase in FOS expression within the paraventricular thalamic nucleus, whilst a significant decrease was observed within the lateroanterior hypothalamic nucleus (S7 Fig). Due to the low expression of FOS, in a relatively dispersed manner within the hypothalamus, the extent to which this QRFP-induced activation was restricted to key individual populations was investigated. NPY- and FOS-expressing neurons were counted in both the arcuate and dorsomedial nuclei. However, QRFP injection did not induce FOS within NPY neurons of either nucleus (S8 Fig). Next, we carried out dual-label immunohistochemistry for FOS and native orexin peptide. Orexin/hypocretin neurons are located within the lateral hypothalamic area and are distinct from nearby QRFP neurons [10]. A single injection of QRFP caused a significant increase in the activity of orexin neurons when compared with saline administration (Fig 5).

**Fig 6:**
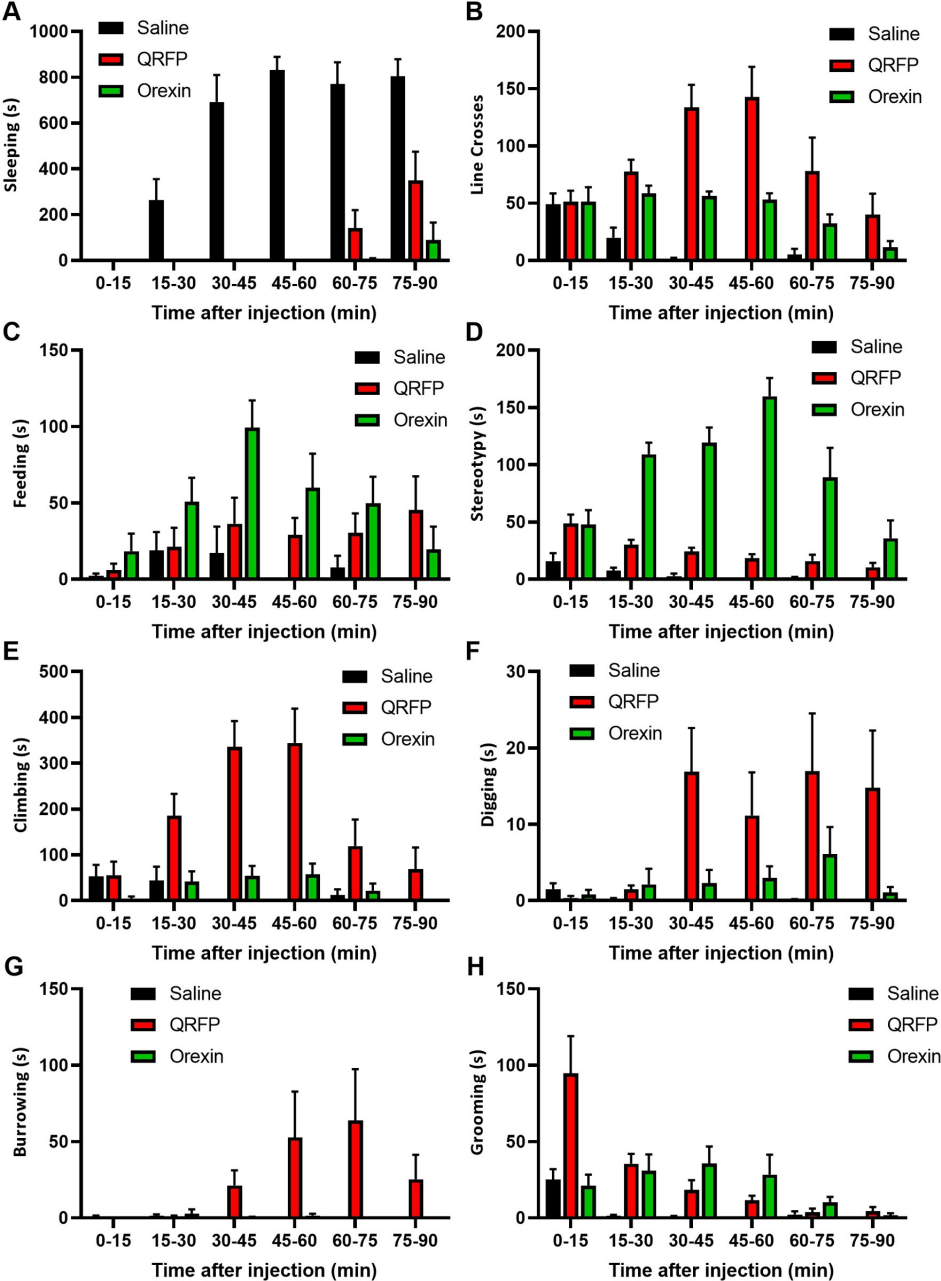
A comparison of the behavioural effects of ICV QRFP and orexin. (A) ICV injection of saline, QRFP or orexin during the day all woke up mice during their normal quiescent period. Saline-treated mice quickly settle down to sleep again. (B) Both peptides increased locomotor activity (line crosses) over the 90-minute observation period. QRFP and orexin had differing effects on behaviours, including (C) feeding, (D) stereotypy, (E) climbing, (F) digging, (G) burrowing and (H) grooming.

We compared the behavioural effects of doses of QRFP and orexin-A peptides (both 7.5 μg), chosen as they produce an equivalent increase in food intake (S9 Fig), though QRFP causes approximately twice as much locomotor activity (line crosses) over a 90 min period (S9 Fig). Mice given a control saline injection two hours into the light phase of the day-night cycle were aroused from sleep for 10–15 min, before settling again (Fig 6A). Both peptides caused increases in locomotor activity over the 90 min following injection (Fig 6B), but QRFP was more potent and the pattern of behaviour was significantly different. Orexin increased feeding behaviour, which peaked within 45 min of injection and then decreased back to baseline over the 90 min observation period (Fig 6C). Orexin-treated mice also displayed short bouts of a stereotypic behaviour (hunched, not moving, often chewing) previously noted by others [22,23], the occurrence of which also peaked at about 45 min after injection (Fig 6D). Although QRFP-treated mice ate an equivalent amount of food, feeding behaviour was less marked, and they did not display any of the same stereotypic behaviour. Mice receiving QRFP maintained locomotor activity and feeding more evenly across the 90 min observation period (Fig 6B and 6C). Notably, QRFP also strongly induced additional foraging behaviours: climbing between 15 and 60 min, and digging and burrowing between 30 and 90 min (Fig 6E–6G). QRFP also increased grooming, a recognised displacement behaviour, in the first 15 min after injection (Fig 6H). Therefore, both peptides increased arousal and locomotor activity. However, while orexin had its strongest effects on consummatory behaviour, QRFP seemed more involved with other appetitive behaviours.

**Fig 7:**
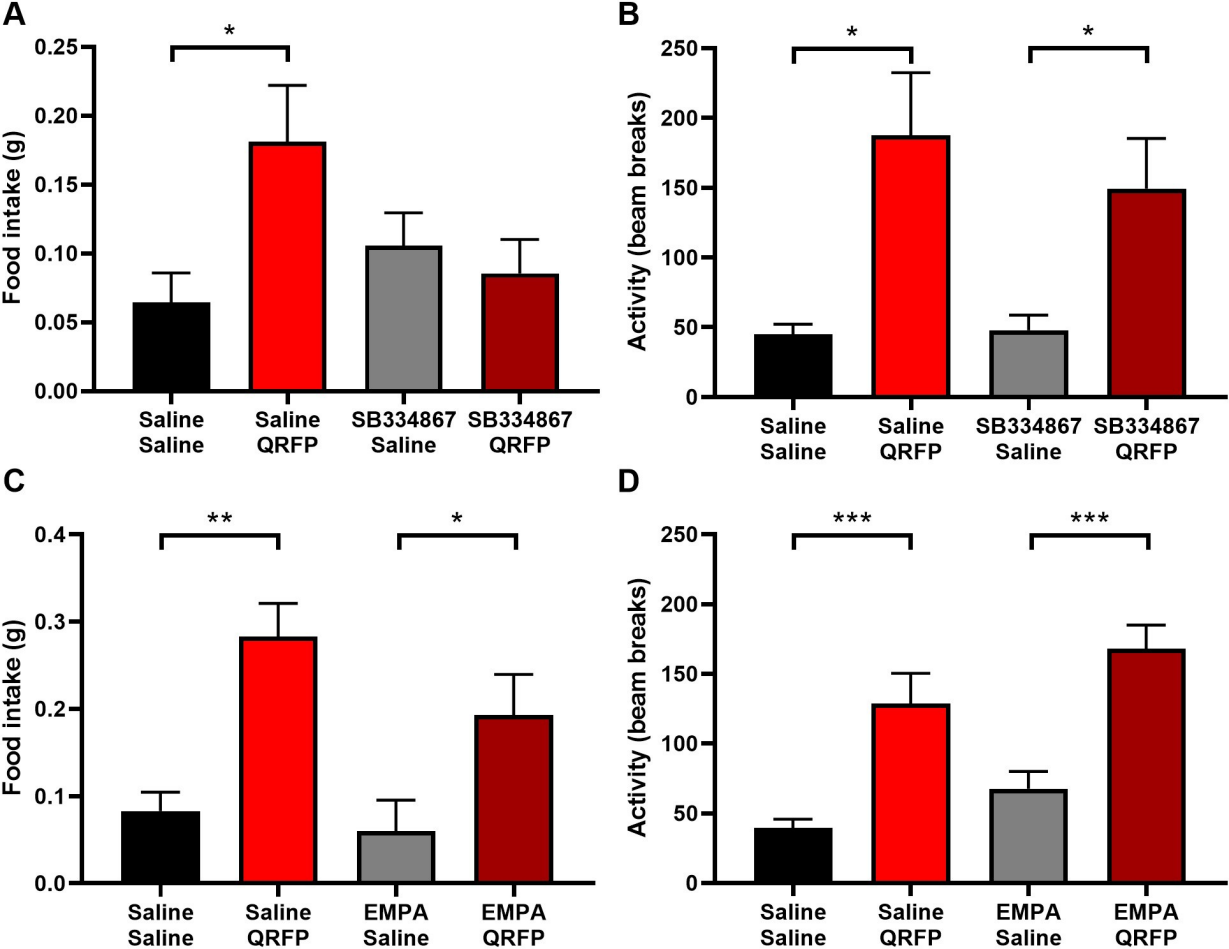
Antagonism of orexin receptor 1 blocks the feeding, but not the locomotor effect of QRFP. Action of orexin receptor 1 antagonist, SB334867, on (A) feeding and (B) locomotor activity induced by QRFP (feeding F_1,32_ = 2.81; activity F_1,32_ = 17.17). (C and D) The orexin receptor 2 antagonist, EMPA, did not affect either parameter (feeding F_1,24_ = 20.7; activity F_1,24_ = 37.65). Two-way ANOVA with Sidak’s *post hoc* test. *p<0.05, **p<0.01, ***p<0.001.

As QRFP activates orexin/hypocretin neurons and there are some similarities in their actions, we asked whether any of the effects of QRFP might be mediated by orexin/hypocretin. The feeding effect of ICV QRFP, but not the effect on locomotor activity, was blocked by IP injection of an orexin/hypocretin receptor 1 antagonist, SB334867 (Fig 7A and 7B). Treatment with an orexin/hypocretin receptor 2 antagonist, EMPA, had no effect on either parameter (Fig 7C and 7D). Taking these results together, QRFP appears to induce orexin-independent appetitive behaviour, and consummatory behaviour perhaps via the activation of orexin/hypocretin neurons and orexin/hypocretin receptor 1.

## Discussion

Germline deletion of the *Qrfp* gene is reported to produce a mild lean phenotype (lower visceral fat), with a 6% reduction in cumulative food intake and no difference in energy expenditure [10]. While there are no obvious effects of *Qrfp* gene knock out on overall locomotor activity or sleep patterns, there is a decrease in awake time during the first six hours of the dark period, suggesting that there may be a role for QRFP in arousal [10]. Importantly, there is reduced centre-directed activity in an open arena and less time spent in the open arms of an elevated-plus maze, both of which might indicate reduced risk avoidance and/or increased exploratory behaviour. This phenotype fits well with our observed effects of the acute administration of QRFP to increase arousal, exploratory and appetitive behaviours and food intake, without a major effect on energy expenditure or interscapular brown adipose tissue temperature. These characteristics are similar with those observed in mice with knock-out of orexin/hypocretin, which have reduced sleep/wake stability, reduced risk avoidance, and normal body weight [17]. By comparison, adult mice in which orexin/hypocretin neurons have been ablated, instead demonstrate hypophagia and increased body weight [18]. Part of the latter phenotype might be explained by complex effects of orexin/hypocretin on energy expenditure [24,25].

Both the 43 amino acid, QRFP, and the shorter form, 26RFa, cause an increase in food intake. Do Rego and colleagues used a series of N-terminally truncated peptides to demonstrate the importance of the C-terminus in the action food intake [26]. For most of our studies, we used the longer QRFP43, as it is the most prevalent form in the mammalian brain [2]. A single injection of QRFP during the day-time phase induced several distinct appetitive behaviours, including locomotor activity, climbing, digging and burrowing. These behaviours occurred along with increased consummatory behaviour, but with no indication that the mice were reaching natural satiety. Increased activity was unaffected by antagonism of orexin/hypocretin receptors. In comparison, mice injected with orexin-A appeared to fixate on consummatory behaviour, including excessive chewing (part of the stereotypy observed by us and others). Orexin can increase food intake by delaying the normal behavioural satiety sequence [23] or by increasing food reward [19], neither of which appears to be the case with QRFP. Thus, it is interestingly that orexin receptor 1 antagonism completely blocked QRFP-induced food intake. These results suggest that QRFP agonism induces both appetitive and consummatory behaviour, but only the latter is dependent on orexin/hypocretin receptor signalling.

Messenger RNAs for both *Gpr103a* and *Gpr103b* are present in multiple regions of the hypothalamus including within the lateral hypothalamic area, paraventricular, ventromedial, dorsomedial and arcuate nuclei [13], which is supported by our binding studies in wild-type, *Gpr103a* knock-out and *Gpr103b* knock-out mice. Although both receptor isoforms appear to be necessary for the appetitive and consummatory effects of QRFP in the brain, currently we have not been able to carry out cell-type specific analysis, which would allow us to determine whether both receptors need to be expressed within the same target neurons. If this is the case, then it is possible that the two receptor isoforms may form heteroreceptor complexes, as may also be the case for combining with other receptors, including potentially with orexin receptors [27]. QRFP receptors are coupled primarily to stimulatory G-proteins Gα_s_ and Gα_q/11_, so might be assumed to cause cellular activation, though other studies have found coupling with inhibitory Gα_i/o_ [28]. We found very little neuronal FOS induction following central administration of QRFP. Notably, QRFP did not induce FOS in NPY neurones of the arcuate or dorsomedial nuclei which would argue against an action through NPY-ergic signalling. However, central QRFP did cause significant activation of orexin/hypocretin neurons in the lateral hypothalamus, which fits with our behavioural results.

Together, our pharmacological studies suggest that QRFP increases arousal and appetitive behaviours, leading to increased food consumption. As the peptide does not increase operant responding, its role is probably to correct negative energy status. Interestingly, we found that the effect of QRFP on food intake could be blocked by an orexin/hypocretin receptor antagonist, which differs to a previous study which reported that QRFP was still effective in orexin/hypocretin knock-out mice [4]. One possible explanation for this discrepancy is that the antagonist blocks the transactivation of Gpr103/orexin receptor dimers by QRFP. Further studies will be required to elucidate potential interplay between these brain signalling systems, and how QRFP neurons, the peptide and its receptors fit into the control of whole-body metabolism.

## Materials and methods

### Animals

All experiments were performed in accordance with the UK Animals (Scientific Procedures) Act 1986, the UK Home Office licence (P9B0614DD) and local ethical review by the University of Manchester Animal Welfare and Ethical Review Body.

Unless stated explicitly, experiments were carried out on adult, male inbred C57Bl/6N mice (20–30 g; Harlan, Blackthorn, UK) or outbred CD1 mice (25–35 g; Charles River, Sandwich UK). *Gpr103a* and *Gpr103b* knock-out mouse lines were sourced from Taconic Biosciences Inc. (Köln, Germany). The *Gpr103a* model was produced by targeted disruption of exon 1 and approximately 400 base pairs of upstream proximal promoter sequence. The *Gpr103a* knock-out model was backcrossed onto C57BL/6NTac to N10 generation equivalence as determined by single nucleotide polymorphism genotyping. The *Gpr103b* model is a conditional knock-out of the gene in C57BL/6NTac mice, in which a floxed exon 3 of the *Gpr103b* was deleted by breeding with a global Cre-recombinase-expressing mouse resulting in deletion of the fourth transmembrane domain and generating a frameshift from exon 2 to a premature stop codon in exon 4. NPY reporter mice were generated by crossing *Npy*-Cre (Mutant Mouse Resource & Research Centre; strain #034810-UCD) and ROSA26-eYFP (B6.129X1-*Gt (ROSA)26Sortm1 (EYFP)Cos*/J; Jackson Laboratories strain #006148). All transgenic strains were maintained as heterozygote colonies (except for homozygous ROSA26-eYFP mice), with regular backcrossing with C57Bl/6N wild-type mice. All offspring were genotyped. Briefly, ear notches were collected from transgenic mice prior to all experimental protocols, and DNA extracted in lysis buffer (Sigma-Aldrich, Gillingham, UK). Polymerase chain reaction (PCR) was used to amplify specific wild-type and target alleles, using unique primers (S3 Table). Reaction mixtures differed for specific PCR reactions, but all contained standard master mix components and GoTaq Hot Start Polymerase (all Promega; Madison, WI, USA).

Mice were housed in the animal unit in constant environmental conditions: 21±2°C and 45±10% humidity on a 12h:12h light-dark cycle (dark phase commencing at 18:00 GMT). All mice were group housed (2–5 per cage) with littermates, with *ad libitum* access to pelleted chow (Special Diet Services; Witham, UK) and sterile pouch water, unless otherwise stated. For some phenotyping experiments, mice were housed singly. Certain phenotyping experiments required mice to be fed a high-energy diet (HED) containing 5.16 kcal/g, where 60% energy comes from fat (58Y1; Test Diets, IPS Ltd., London, UK).

### Tissue distribution

To look for whole-body tissue distribution of the receptors, DNase-treated RNA was obtained from BioChain Institute (Newark, CA, USA) or from C57Bl/6N mouse tissues using TRIzol reagent (Invitrogen, Carlsbad, CA) and reverse transcribed using the SuperScript III First-Strand synthesis kit (Invitrogen) to obtain cDNA. cDNA was amplified using TaqMan universal PCR master mix and gene specific primer probes using a 7900HT Sequence Detection System (all Applied Biosystems, Thermo Fisher Scientific; Wilmington, DE, USA) and SDS2.3 software. Gene expression was normalized to copies of GAPDH using the formula: (2^(Ct GAPDH–Ct gene of interest)) X 10^6. For tissue specific cDNA panels, cDNA was custom plated by Origene in 384 well plates (Applied Biosystems).

To confirm receptor deletion in the *Gpr103a* and *Gpr103b* lines, selected tissues from wild-type and knock-out littermates were dissected and RNA reverse transcribed as above. End-point, reverse transcription-PCR was carried out using selective primers (S3 Table). To demonstrate selective loss of binding, C57Bl/6N, *Gpr103a* knock-out and *Gpr103b* knock-out mice were euthanized by decapitation and their brains dissected and frozen on dry ice. Twelve μm frozen coronal sections were thaw-mounted onto chrome alum/gelatin-coated slides and stored at -70°C. Sections were pre-incubated at 25°C for 30 min in buffer (20mM Tris-HCl, 5mM Mg acetate, 0.25% BSA). Incubations were conducted in the same buffer containing 0.3 nM ^125^I-QRFP (specific activity 2200 Ci/mmol; ViTrax, Placentia, CA, USA) at 25°C for 90 min. Non-specific binding was determined on adjacent sections in the presence of 1 μM human QRFP43 (CPC, Sunnyvale, CA, USA). Sections were washed twice on ice (2X 10 min) in pre-incubation buffer without BSA, followed by a rinse in ice-cold distilled water. Slides were dried under a cool stream of air and apposed to a Fuji phosphorimaging plate (BAS-IP TR 2025) for 18–24 hours. Plates were scanned using a BAS-5000 imager (Fuji, Tokyo, Japan).

### Phenotyping and behavioural studies

To allow intracerebroventricular (ICV) injections, mice were unilaterally implanted with guide cannulae into the right lateral ventricle (0.4 mm caudal, 1.0 mm lateral to bregma), under anaesthesia with 3% isofluorane (Abbot Abbvie Ltd, Maidenhead, UK) in oxygen (1500 ml/min). Single ICV injections of QRFP43, 26RFa and orexin-A (all Phoenix Pharmaceuticals, Burlingame, CA, USA), were made in 3 μl total volume. Single intraperitoneal (IP) injections of the orexin receptor 1 and 2 antagonists, SB334867 and N-ethyl-2-[(6-methoxy-pyridin-3-yl)-(toluene-2-sulphonyl)-amino]-N-pyridin-3-ylmethyl-acetamide (EMPA) (both Tocris Bio-Techne Ltd., Abingdon, UK) were made in normal saline. Food intake and basic locomotor activity experiments were carried out on separate cohorts of mice in their home cages intersected by infrared beams. To measure behaviour more closely, mice were videoed for 90 min after injection and assessed by a blinded experimenter. In addition to food intake and locomotor activity (line breaks triggered when the centre mass of the animal crosses line), time spent sleeping (curled up with eyes closed), ingesting food (holding food in paws and/or chewing), grooming (wiping, scratching or rubbing), climbing, digging and burrowing were recorded. Mice injected with orexin also displayed some stereotypic behaviour (see text and references for description). For operant response testing, a cohort of male CD1 mice were trained in an operant responding paradigm similar with that previously described [19]. Throughout the initial training stages, singly housed mice were food restricted, on around 60% of normal food intake, to maintain a body weight at 85% of their pre-study weight. Lever presses and rewards (Yazoo strawberry milkshake; FrieslandCampina, Amersfoot, Netherlands) earned were monitored using a hardware and ABETII software from Lafayette Instruments (Campden Instruments, Loughborough, UK) to identify the correct time for training progression through a fixed-ratio schedule and then a progressive-ratio (PR) schedule. When mice were stably responding to the PR schedule, as determined by less than 15% variability between three consecutive days’ breakpoint, training was complete and the testing phase began.

Energy expenditure (oxygen consumption) and visits to the food hopper were measured in mice acclimated to indirect calorimetry cages (Columbus Instruments, Columbus, OH, USA). To measure the effect of QRFP or 26RFa on brown adipose tissue (BAT) temperature, two separate cohorts of male mice (shaved between the scapula) had food withheld at lights-on to remove confounding effects of a recent thermogenic response to feeding. Interscapular BAT temperature was measured indirectly using a thermal camera (FLIR; Wilsonville, OR, USA). Mice were recorded in their home cage and videos were analysed using ResearchIR software (FLIR) with correct environmental parameters incorporated into its algorithms: ambient temperature, humidity and distance to mouse. For the oral glucose tolerance test, blood-glucose levels were measured from the tail vein using a glucose monitor (Accuchek, Roche Diagnostics Ltd, Burgess Hill, UK). Baseline measures were taken immediately prior to ICV injection of peptide and by oral gavage of glucose (2 g/ml at 4 ml/kg dose volume; Fisher Scientific, Loughborough, UK).

### Immunohistochemistry

Fluorescence-based, dual-label immunohistochemistry was carried out on free-floating mouse brain sections. All washing and incubation steps detailed below were carried out with gentle agitation of the multi-well plates containing sections. Grouped brain sections were washed thoroughly in phosphate buffer containing 0.2% v/v Triton X-100 (Sigma-Aldrich, Gillingham, UK) followed by an hour-long ‘blocking’ incubation in 10% normal serum. The serum was from the same species from which the secondary antibody was derived. Sections were incubated in the relevant primary antibody overnight at 4°C, in 1% normal serum. Sections then were washed thoroughly in phosphate buffer, prior to incubation with appropriate secondary antibodies. Secondary antibody incubation was carried out for two hours in 5% normal serum at room temperature. Final washes were carried out in phosphate buffer followed by a single wash in distilled water prior to mounting. Slides were left overnight to dry, and covered with Prolong Gold (Invitrogen; Carlsbad, CA, USA) and a coverslip. Primary antibodies were against FOS (SC-52; Santa Cruz), orexin/hypocretin (SC-8070; Santa Cruz Biotechnology, Dallas, TX, USA) and GFP (13970; Cell Signalling). Secondary antibodies were: donkey anti-rabbit^BIOTIN^ (711-065-152; Jackson ImmunoResearch, Ely, UK) followed by streptavidin^594^ (016-580-084; Jackson) for FOS; donkey anti-goat^488^ (705-545-003; Jackson) for orexin/hypocretin; donkey anti-chicken^488^ (703-545-155; Jackson) for hrGFP or eYFP. For single-label FOS immunohistochemistry, instead of fluorescent secondary antibody, the primary was followed by incubation biotinylated secondary antibody, streptavidin-biotin-peroxidase complex and, finally, with a diaminobenzidine kit containing nickel (all Vector Laboratories, Peterborough, UK).

### Statistics

Statistical comparisons between groups were analysed using either unpaired two-tailed Student’s t-tests or two-way analysis of variance with either *post hoc* Sidak’s tests or Dunnett’s tests, as indicated. Differences were considered significant if p < 0.05. The values of n and statistical significance are reported in the Figures legends.

## Supporting information

S1 Fig A comparison of the effects of QRFP, PrRP and NPFF on food intake. Peptides were injected ICV into outbred CD1 mice just before lights out, to measure normal night-time feeding one hour after injection. (A) NPFF 2, 4 and 8 nmol (F_3,16_ = 6.31). (B) PrRP 1, 2 and 4 nmol (F_3,19_ = 6.11). (C) QRFP 1, 5 and 10 μg (F_3,18_ = 4.44). (10 μg = 2.2 nmol). One-way ANOVA with Dunnett’s *post hoc* test. *p<0.05 **p<0.01.
(PDF)

S2 Fig QRFP and 26RFa do not affect brown adipose-dependent adaptive thermogenesis acutely. Interscapular BAT temperature was measured in C57Bl/6N mice by infrared thermography before and 2 h after injection. There was no effect after (A) QRFP or (B) 26RFa injection. Results analysed by a two-way ANOVA.
(PDF)

S3 Fig Effects of orexin and QRFP on operant responding. CD1 mice were injected ICV with orexin (7 μg) QRFP (2 μg or 5 μg, ICV). Orexin increased (A) breakpoint and (B) correct lever presses (breakpoint t_7_ = 2.95; lever presses t_7_ = 3.98). (C and D) QRFP had no effect on operant responding (breakpoint t_6_ = 0.57; lever presses t_6_ = 0.71). *p<0.05 paired t-test.
(PDF)

S4 Fig Tissue distributions of QRFP receptors. Normalized expression of (A) human *GPR103* and (B) mouse *Gpr103a* and *Gpr103b* in cDNA tissue panels.
(PDF)

S5 Fig Tissue distributions of QRFP receptors in *Gpr103a* and *Gpr103b* knock-out mice. Normalized expression of (A) *Gpr103a* and (B) *Gpr103b* in cDNA tissue panels. Mice I-III are wild-type and mice IV-VI are knock out for each gene studied in the following tissues: Brown adipose tissue (a), eye (b), hypothalamus (c), liver (d), skeletal muscle (e), pancreas (f), epididymal white adipose (g) and inguinal white adipose (h).
(PDF)

S6 Fig Examples of ^125^I-QRFP binding on mouse brain sections. Autoradiograms of [^125^I]QRFP43 binding in wildtype (WT), *Gpr103a* knock-out (*Gpr103a* KO), and *Gpr103b* knock-out (*Gpr103b* KO) mouse brain. Sections are representative from n = 3 brains from each strain. (A) olfactory bulb external plexiform layer, (B) olfactory bulb internal plexiform layer, (C) molecular layer of cerebral cortex, (D) caudate/putamen, (E) nucleus accumbens shell, (F) claustrum/dorsal endopiriform nucleus, (G) subfornical organ, (H) reuniens thalamic nucleus, (I) hypothalamus, (J) piriform cortex, (K) amygdala, (L) granule cell layer of cerebral cortex, (M) Edingar-Westphal nucleus, (N) hippocampus, (O) interpeduncular nucleus, (P) spinal 5 nucleus, (Q) hypoglossal nucleus
(PDF)

S7 Fig Effect of QRFP on FOS expression in the mouse forebrain. (A) Third ventricular QRFP increased FOS expression in the paraventricular nucleus of the thalamus (PVT) and decreased it in the lateroanterior hypothalamic nucleus (LA), but did not have any significant effect on FOS in other, anatomically defined hypothalamic nuclei. *p<0.05, unpaired t-test. Arc, arcuate nucleus; DMN, dorsomedial nucleus; LHA, lateral hypothalamic area; PVN, paraventricular nucleus; SCN, suprachiasmatic nucleus; VMN, ventromedial nucleus. Representative images of the lateroanterior hypothalamus, LA, following injection of (B) saline vehicle or (C) QRFP. Representative images of the paraventricular nucleus of the thalamus, PVT, following injection of (D) saline vehicle or (E) QRFP. D3V, dorsal third ventricle.
(PDF)

S8 Fig QRFP does not activate NPY neurons in either the dorsomedial or arcuate nuclei. Representative images of dual-fluorescence immunohistochemistry for FOS (magenta) and GFP in the (A and B) dorsomedial nucleus and (C and D) arcuate nucleus of *Npy*-Cre::eYFP mice, injected with either (A and C) saline vehicle or (B and D) QRFP. 3V, third ventricle.
(PDF)

S9 Fig A comparison of QRFP and orexin on food intake and locomotor activity. (A) ICV injection of QRFP or orexin caused a similar increase in food intake over a 90 min observation period (F_2,18_ = 8.09). (B) ICV injection of QRFP and orexin increased locomotor activity (line crosses); the increase following QRFP was almost double that following orexin (F_2,16_ = 16.89). One-way repeated measures ANOVA with Sidak’s *post hoc* test. *p<0.05, **p<0.01, ***p<0.001.
(PDF)

S1 Table Homology search on Gpr103 from different species. (PDF)

S2 Table Additional phenotypic data for Gpr103a and Gpr103b knock-out mice. All comparisons are between male, homozygous wild-type and knock-out littermates. No differences in feeding parameters or in an oral glucose tolerance test were noted between genotypes.
(PDF)

S3 Table Primers used for genotyping. (PDF)

S1 Data (PZFX)

S2 Data (PZFX)
