# Supplementary material for: The hypothalamic RFamide, QRFP, increases feeding and locomotor activity: The role of Gpr103 and orexin receptors
Source: PLoS One. 2022 Oct 17;17(10):e0275604. doi: 10.1371/journal.pone.0275604 (PMC9576062; doi:10.1371/journal.pone.0275604)
Supplement: S1 Fig — Peptides were injected ICV into outbred CD1 mice just before lights out, to measure normal night-time feeding one hour after injection. (A) NPFF 2, 4 and 8 nmol (F3,16 = 6.31). (B) PrRP 1, 2 and 4 nmol (F3,19 = 6.11). (C) QRFP 1, 5 and 10 μg (F3,18 = 4.44). (10 μg = 2.2 nmol). One-way ANOVA with Dunnett’s post hoc test. *p<0.05 **p<0.01. (PDF) [file pone.0275604.s001.pdf]

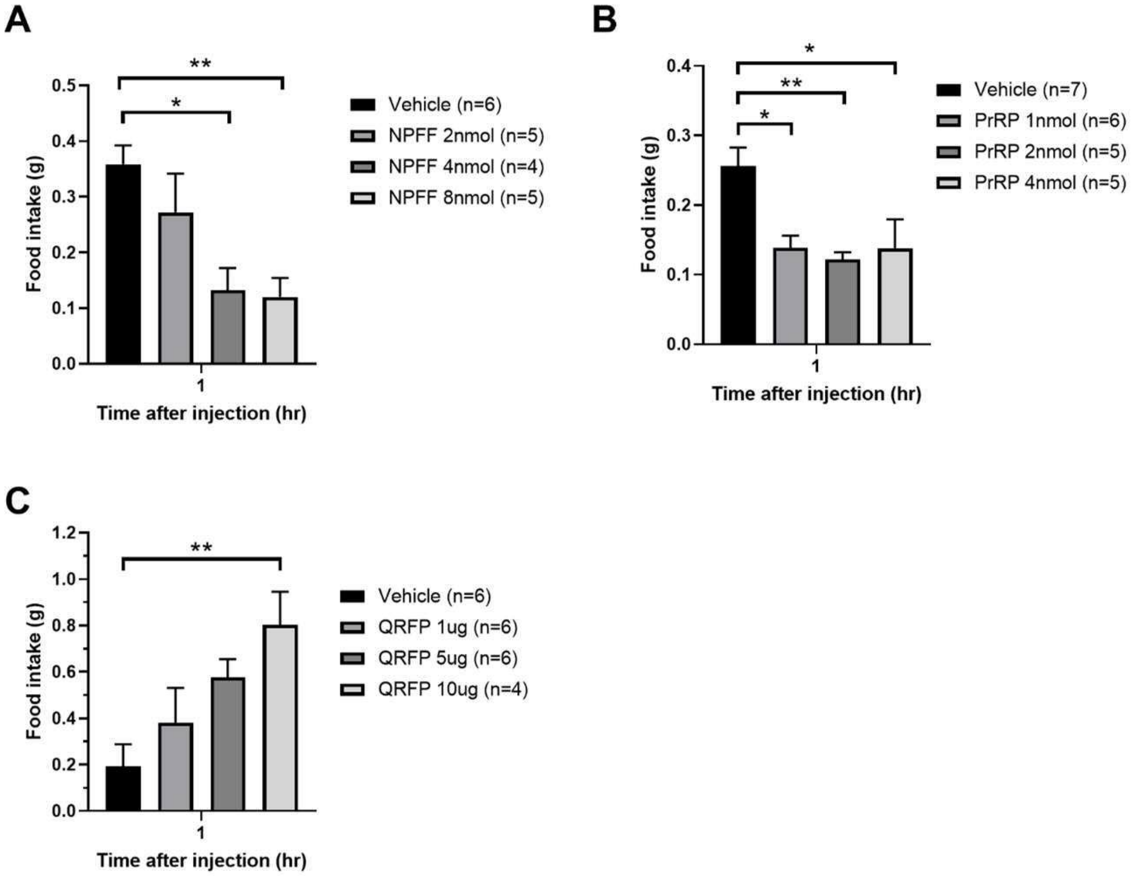

S1 Fig. A comparison of the effects of QRFP, PrRP and NPFF on food intake. Peptides were injected ICV into outbred CD1 mice just before lights out, to measure normal night-time feeding one hour after injection. (A) NPFF 2, 4 and 8 nmol ($F_{3,16}$ = 6.31). (B) PrRP 1, 2 and 4 nmol ($F_{3,19}$ = 6.11). (C) QRFP 1, 5 and 10 µg ($F_{3,18}$ = 4.44). (10 µg = 2.2 nmol). One-way ANOVA with Dunnett's *post hoc* test. *p<0.05 **p<0.01.
