# Supplementary material for: The hypothalamic RFamide, QRFP, increases feeding and locomotor activity: The role of Gpr103 and orexin receptors
Source: PLoS One. 2022 Oct 17;17(10):e0275604. doi: 10.1371/journal.pone.0275604 (PMC9576062; doi:10.1371/journal.pone.0275604)
Supplement: S2 Fig — Interscapular BAT temperature was measured in C57Bl/6N mice by infrared thermography before and 2 h after injection. There was no effect after (A) QRFP or (B) 26RFa injection. Results analysed by a two-way ANOVA. (PDF) [file pone.0275604.s002.pdf]

A

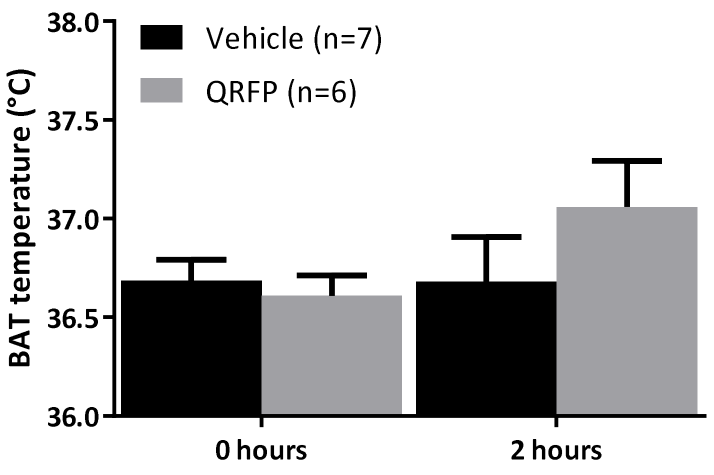

B

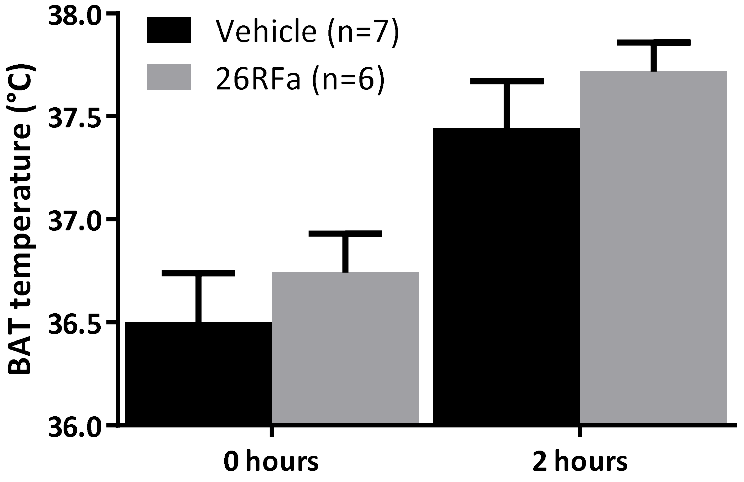

S2 Fig. QRFP and 26RFa do not affect thermogenesis acutely. Interscapular BAT temperature was measured in C57/bl6 mice by infrared thermography. There was no effect after (A) QRFP or (B) 26RFa injection. Results analysed by a two-way ANOVA
