# Supplementary material for: The hypothalamic RFamide, QRFP, increases feeding and locomotor activity: The role of Gpr103 and orexin receptors
Source: PLoS One. 2022 Oct 17;17(10):e0275604. doi: 10.1371/journal.pone.0275604 (PMC9576062; doi:10.1371/journal.pone.0275604)
Supplement: S3 Fig — CD1 mice were injected ICV with orexin (7 μg) QRFP (2 μg or 5 μg, ICV). Orexin increased (A) breakpoint and (B) correct lever presses (breakpoint t7 = 2.95; lever presses t7 = 3.98). (C and D) QRFP had no effect on operant responding (breakpoint t6 = 0.57; lever presses t6 = 0.71). *p<0.05 paired t-test. (PDF) [file pone.0275604.s003.pdf]

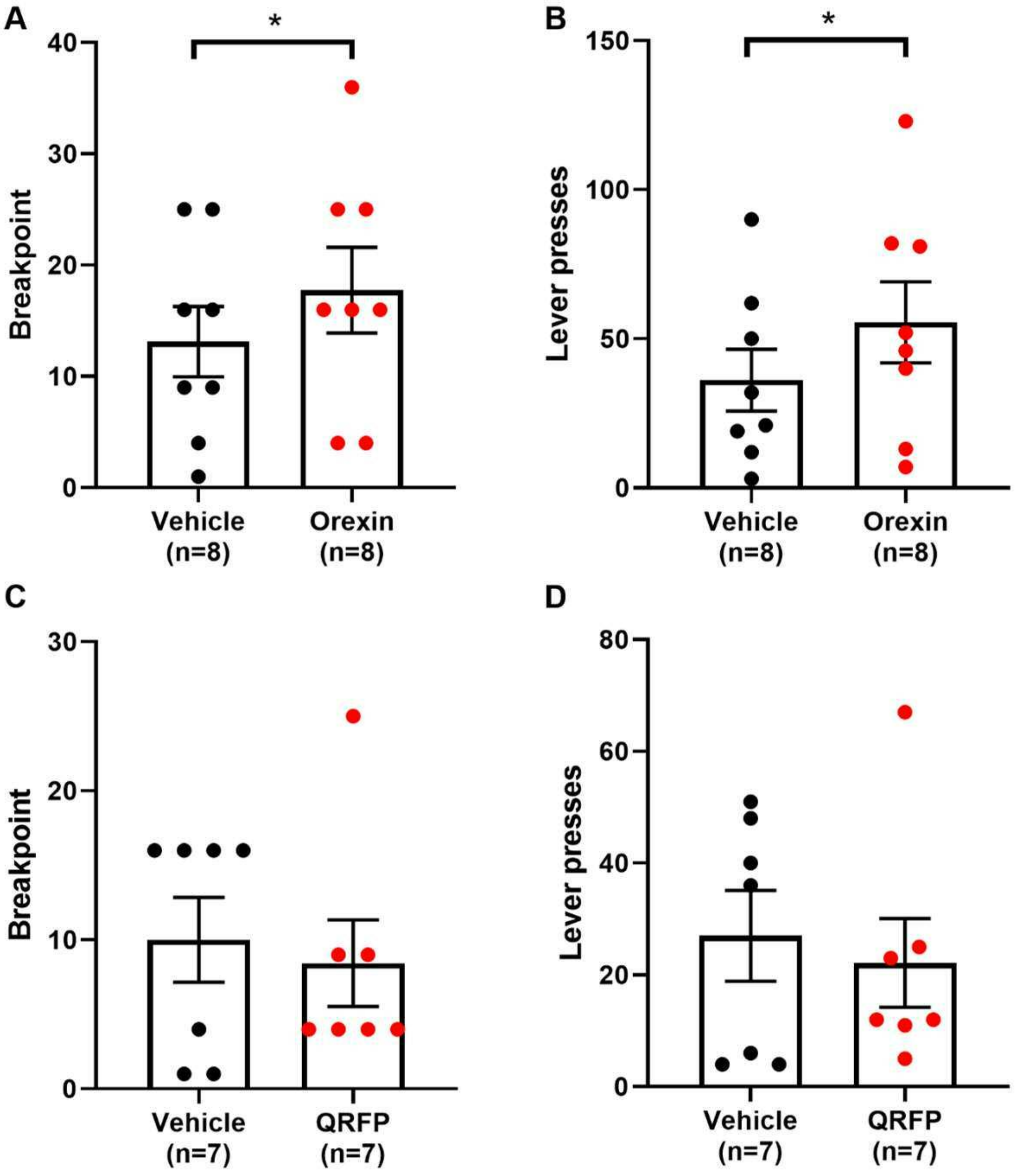

S3 Fig. Effects of orexin and QRFP on operant responding. CD1 mice were injected ICV with orexin (7 µg) QRFP (2 µg or 5 µg, ICV). Orexin increased (A) breakpoint and (B) correct lever presses (breakpoint $t_7 = 2.95$; lever presses $t_7 = 3.98$). (C and D) QRFP had no effect on operant responding (breakpoint $t_6 = 0.57$; lever presses $t_6 = 0.71$). $^*p<0.05$ paired t-test.
