# Supplementary material for: The hypothalamic RFamide, QRFP, increases feeding and locomotor activity: The role of Gpr103 and orexin receptors
Source: PLoS One. 2022 Oct 17;17(10):e0275604. doi: 10.1371/journal.pone.0275604 (PMC9576062; doi:10.1371/journal.pone.0275604)
Supplement: S4 Fig — Normalized expression of (A) human GPR103 and (B) mouse Gpr103a and Gpr103b in cDNA tissue panels. (PDF) [file pone.0275604.s004.pdf]

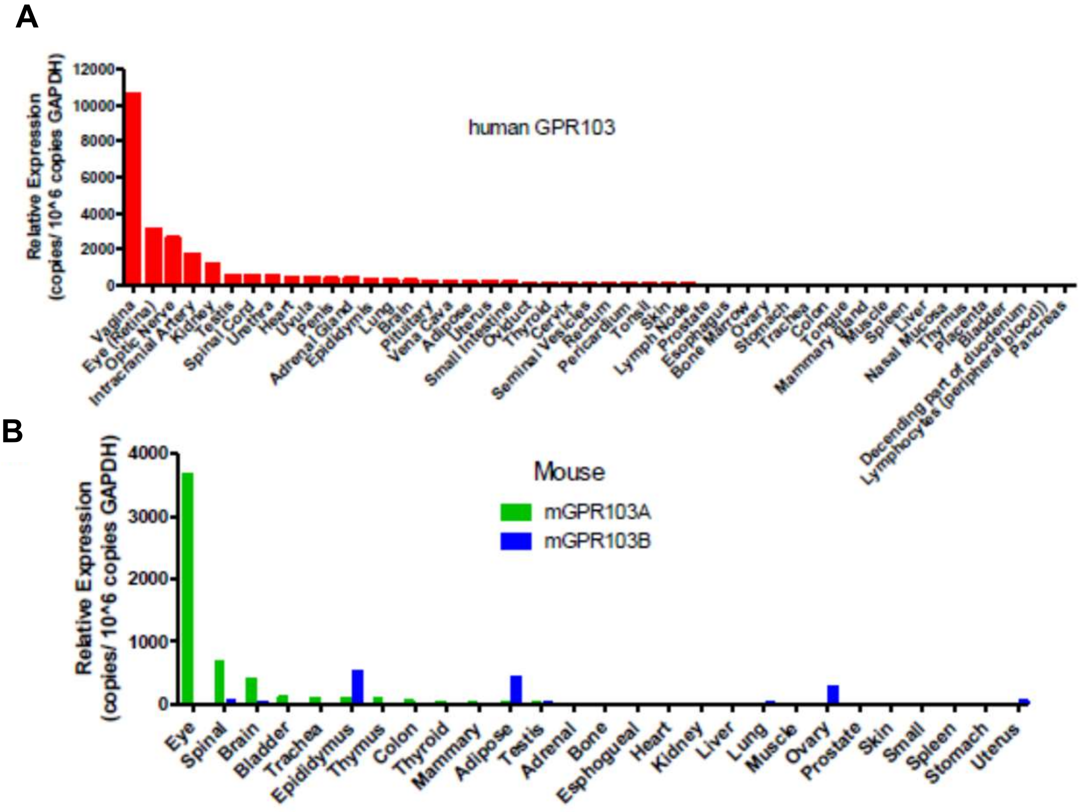

S4 Fig. Tissue distributions of QRFP receptors. Normalized expression of (A) human GPR103 and (B) mouse Gpr103a and Gpr103b in cDNA tissue panels.
