# Supplementary material for: The hypothalamic RFamide, QRFP, increases feeding and locomotor activity: The role of Gpr103 and orexin receptors
Source: PLoS One. 2022 Oct 17;17(10):e0275604. doi: 10.1371/journal.pone.0275604 (PMC9576062; doi:10.1371/journal.pone.0275604)
Supplement: S5 Fig — Normalized expression of (A) Gpr103a and (B) Gpr103b in cDNA tissue panels. Mice I-III are wild-type and mice IV-VI are knock out for each gene studied in the following tissues: Brown adipose tissue (a), eye (b), hypothalamus (c), liver (d), skeletal muscle (e), pancreas (f), epididymal white adipose (g) and inguinal white adipose (h). (PDF) [file pone.0275604.s005.pdf]

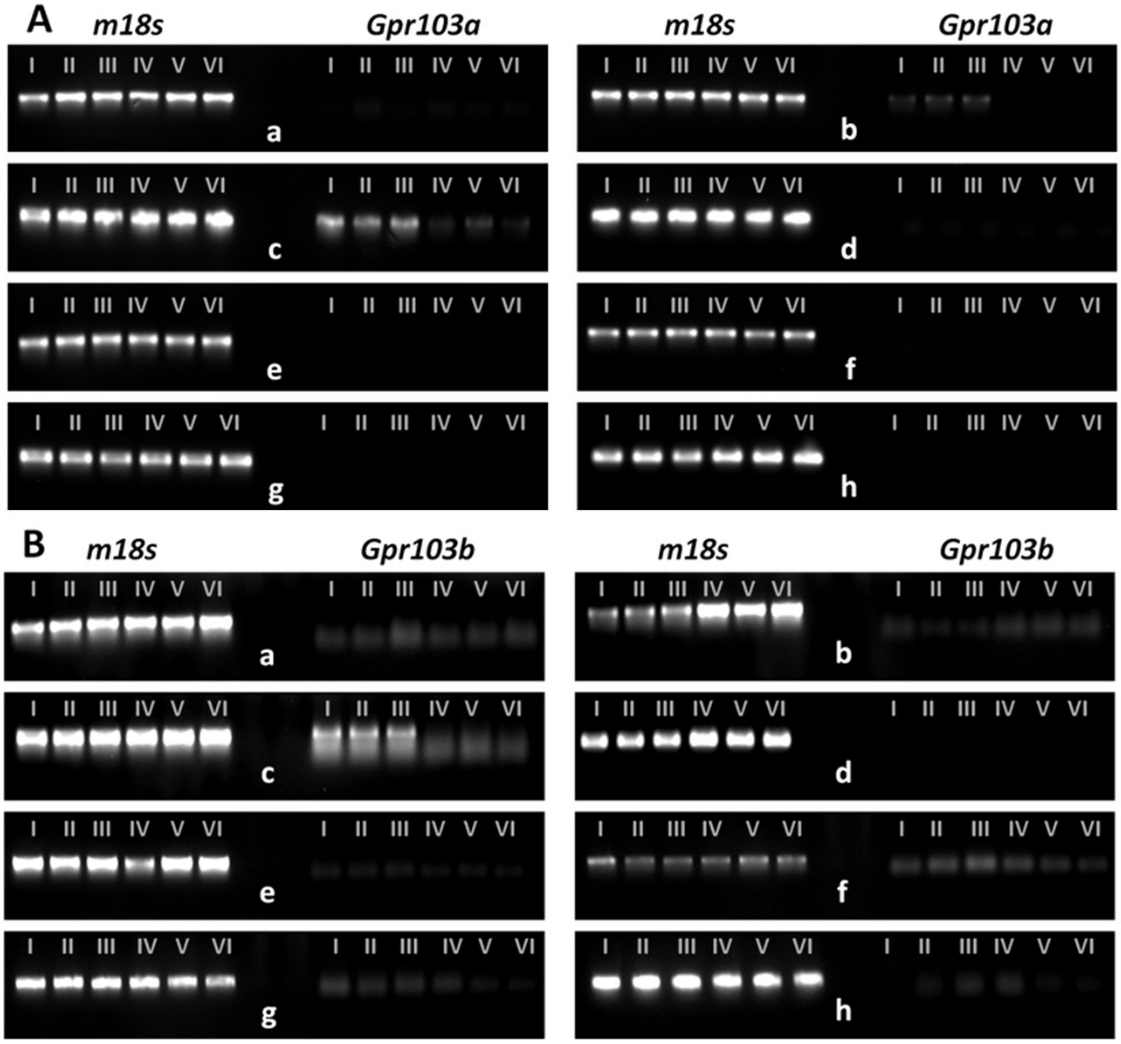

S5 Fig. Tissue distributions of QRFP receptors in *Gpr103a* and *Gpr103b* knock-out mice. Normalized expression of (A) *Gpr103a* and (B) *Gpr103b* in cDNA tissue panels. Mice I-III are wild-type and mice IV-VI are knock out for each gene studied in the following tissues: brown adipose tissue (a), eye (b), hypothalamus (c), liver (d), skeletal muscle (e), pancreas (f), epididymal white adipose (g) and inguinal white adipose (h).
