# Supplementary figures and images for: The hypothalamic RFamide, QRFP, increases feeding and locomotor activity: The role of Gpr103 and orexin receptors
Source: PLoS One. 2022 Oct 17;17(10):e0275604. doi: 10.1371/journal.pone.0275604 (PMC9576062; doi:10.1371/journal.pone.0275604)

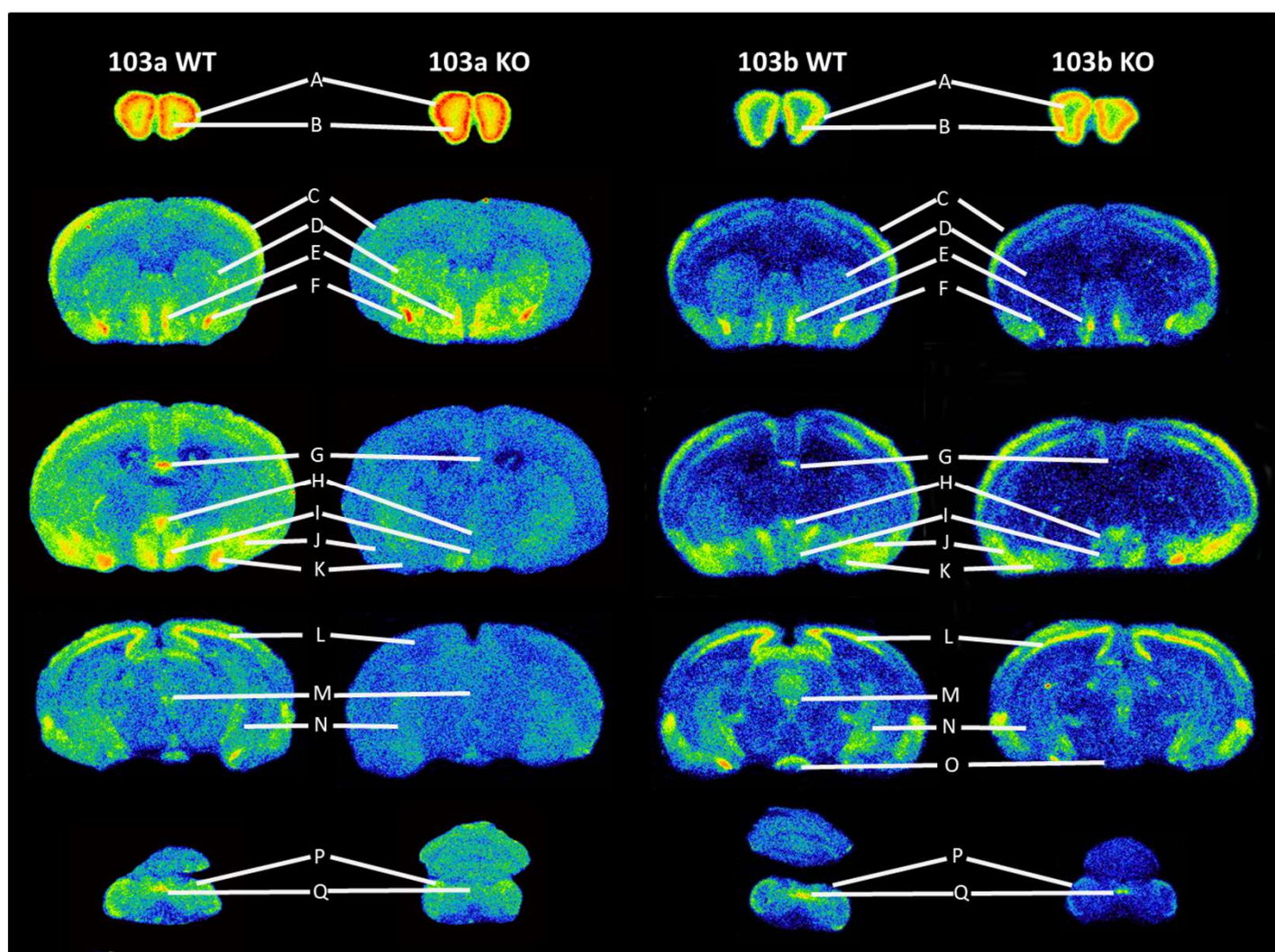

**S6 Fig. Examples of  $^{125}\text{I}$ -QRFP binding on mouse brain sections**

Supplement: S6 Fig — Autoradiograms of [125I]QRFP43 binding in wildtype (WT), Gpr103a knock-out (Gpr103a KO), and Gpr103b knock-out (Gpr103b KO) mouse brain. Sections are representative from n = 3 brains from each strain. (A) olfactory bulb external plexiform layer, (B) olfactory bulb internal plexiform layer, (C) molecular layer of cerebral cortex, (D) caudate/putamen, (E) nucleus accumbens shell, (F) claustrum/dorsal endopiriform nucleus, (G) subfornical organ, (H) reuniens thalamic nucleus, (I) hypothalamus, (J) piriform cortex, (K) amygdala, (L) granule cell layer of cerebral cortex, (M) Edingar-Westphal nucleus, (N) hippocampus, (O) interpeduncular nucleus, (P) spinal 5 nucleus, (Q) hypoglossal nucleus (PDF) [file pone.0275604.s006.pdf]
