# Supplementary material for: The hypothalamic RFamide, QRFP, increases feeding and locomotor activity: The role of Gpr103 and orexin receptors
Source: PLoS One. 2022 Oct 17;17(10):e0275604. doi: 10.1371/journal.pone.0275604 (PMC9576062; doi:10.1371/journal.pone.0275604)
Supplement: S7 Fig — (A) Third ventricular QRFP increased FOS expression in the paraventricular nucleus of the thalamus (PVT) and decreased it in the lateroanterior hypothalamic nucleus (LA), but did not have any significant effect on FOS in other, anatomically defined hypothalamic nuclei. *p<0.05, unpaired t-test. Arc, arcuate nucleus; DMN, dorsomedial nucleus; LHA, lateral hypothalamic area; PVN, paraventricular nucleus; SCN, suprachiasmatic nucleus; VMN, ventromedial nucleus. Representative images of the lateroanterior hypothalamus, LA, following injection of (B) saline vehicle or (C) QRFP. Representative images of the paraventricular nucleus of the thalamus, PVT, following injection of (D) saline vehicle or (E) QRFP. D3V, dorsal third ventricle. (PDF) [file pone.0275604.s007.pdf]

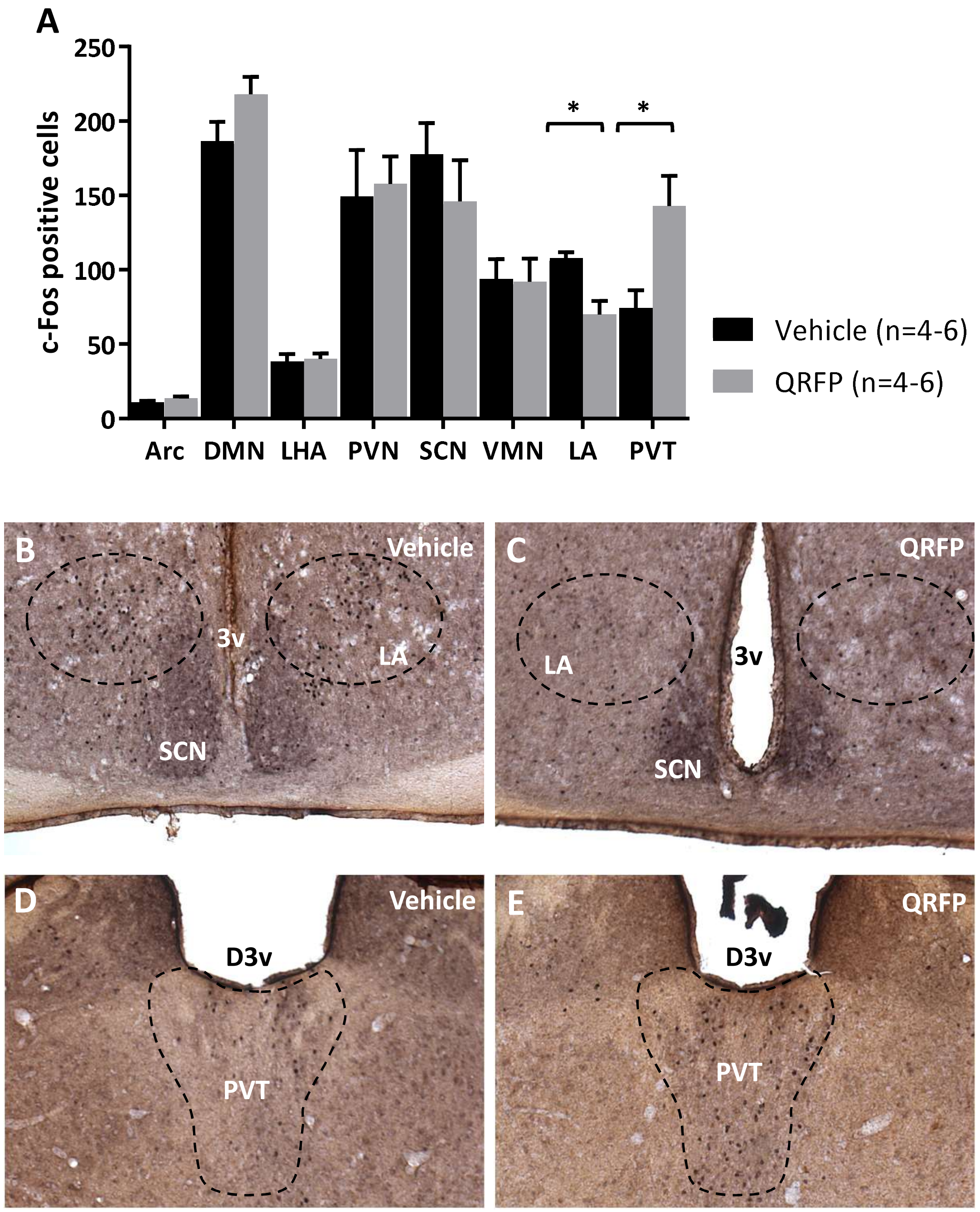

S7 Fig. Effect of QRFP on FOS expression in the mouse forebrain. (A) Third ventricular QRFP increased FOS expression in the paraventricular nucleus of the thalamus (PVT) and decreased it in the lateroanterior hypothalamic nucleus (LA), but did not have any significant effect on FOS in other, anatomically defined hypothalamic nuclei. *$P<0.05$, unpaired t-test. Arc, arcuate nucleus; DMN, dorsomedial nucleus; LHA, lateral hypothalamic area; PVN, paraventricular nucleus; SCN, suprachiasmatic nucleus; VMN, ventromedial nucleus. Representative images of the lateroanterior hypothalamus, LA, following injection of (B) saline vehicle or (C) QRFP. Representative images of the paraventricular nucleus of the thalamus, PVT, following injection of (D) saline vehicle or (E) QRFP. D3V, dorsal third ventricle.
