# Supplementary material for: The hypothalamic RFamide, QRFP, increases feeding and locomotor activity: The role of Gpr103 and orexin receptors
Source: PLoS One. 2022 Oct 17;17(10):e0275604. doi: 10.1371/journal.pone.0275604 (PMC9576062; doi:10.1371/journal.pone.0275604)
Supplement: S8 Fig — Representative images of dual-fluorescence immunohistochemistry for FOS (magenta) and GFP in the (A and B) dorsomedial nucleus and (C and D) arcuate nucleus of Npy-Cre::eYFP mice, injected with either (A and C) saline vehicle or (B and D) QRFP. 3V, third ventricle. (PDF) [file pone.0275604.s008.pdf]

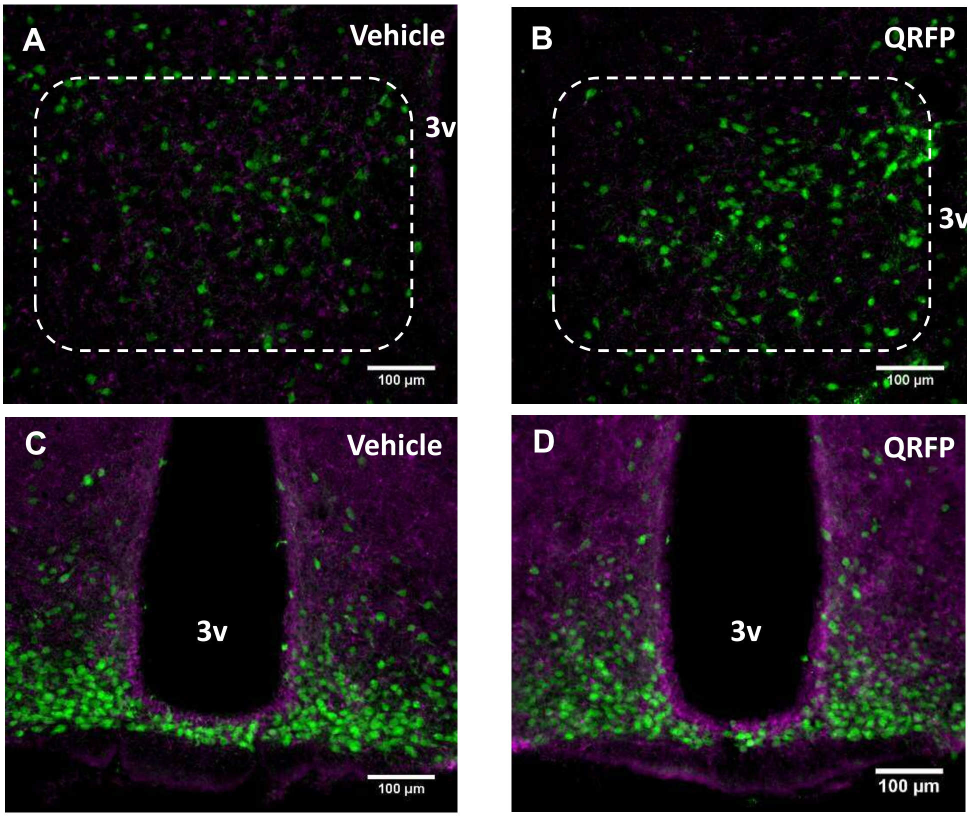

S8 Fig. QRFP does not activate NPY neurons in either the dorsomedial or arcuate nuclei. Representative images of dual-fluorescence immunohistochemistry for FOS (magenta) and GFP in the (A and B) dorsomedial nucleus and (C and D) arcuate nucleus of *Npy*-Cre::eYFP mice, injected with either (A and C) saline vehicle or (B and D) QRFP. 3V, third ventricle.
