# Supplementary material for: The hypothalamic RFamide, QRFP, increases feeding and locomotor activity: The role of Gpr103 and orexin receptors
Source: PLoS One. 2022 Oct 17;17(10):e0275604. doi: 10.1371/journal.pone.0275604 (PMC9576062; doi:10.1371/journal.pone.0275604)
Supplement: S9 Fig — (A) ICV injection of QRFP or orexin caused a similar increase in food intake over a 90 min observation period (F2,18 = 8.09). (B) ICV injection of QRFP and orexin increased locomotor activity (line crosses); the increase following QRFP was almost double that following orexin (F2,16 = 16.89). One-way repeated measures ANOVA with Sidak’s post hoc test. *p<0.05, **p<0.01, ***p<0.001. (PDF) [file pone.0275604.s009.pdf]

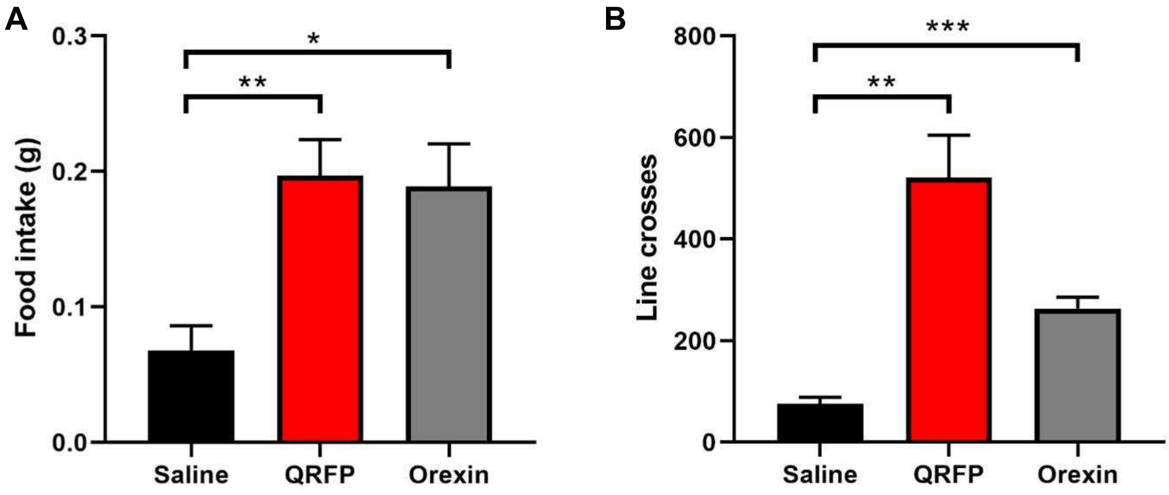

S9 Fig. A comparison of QRFP and orexin on food intake and locomotor activity. (A) ICV injection of QRFP or orexin caused a similar increase in food intake over a 90 min observation period ($F_{2,18} = 8.09$). (B) ICV injection of QRFP and orexin increased locomotor activity (line crosses); the increase following QRFP was almost double that following orexin ($F_{2,16} = 16.89$). One-way repeated measures ANOVA with Sidak's *post hoc* test. *$p<0.05$, **$p<0.01$, ***$p<0.001$.
