# Supplementary material for: The hypothalamic RFamide, QRFP, increases feeding and locomotor activity: The role of Gpr103 and orexin receptors
Source: PLoS One. 2022 Oct 17;17(10):e0275604. doi: 10.1371/journal.pone.0275604 (PMC9576062; doi:10.1371/journal.pone.0275604)
Supplement: S1 Table — (PDF) [file pone.0275604.s010.pdf]

A

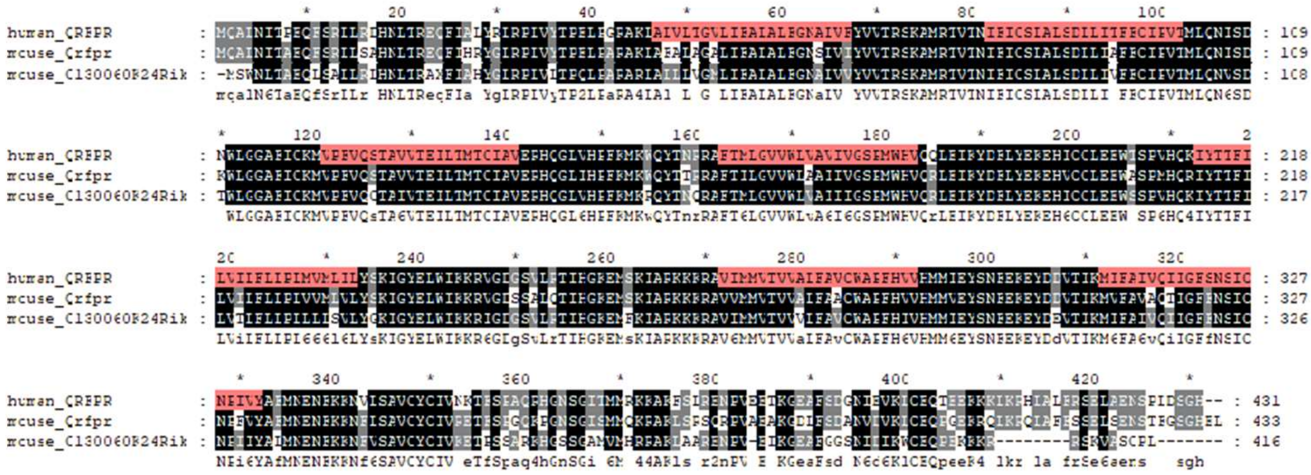

B

SPECIES	GENE	NAME	CHROMOSOME	ACCESSION #	% IDENTITY TO HUMAN	% SIMILARITY TO HUMAN
Human	QRFPR	GPR103	4	NM_198179.2		
Cynomolgus monkey	QRFPR	GPR103	5	FR874248.1	98	100
Dog	QRFPR	GPR103	19	chr: BROADD2:19: 21716088: 21785582	90	94
Mouse	QRFPR	GPR103A	3	AK034431.1	83	90
	C130060K24Rik	GPR103B	6	NM_175524.4	77	87
Rat	QRFPR	GPR103A	2	NM198199.1	83	90
	RGD1560028	GPR103B	4	NM_001109239.1	78	86
Hamster	QRFPR	GPR103A		NW_003615011	82*	91*
		GPR103B		NW_003614267	76*	85*

*Gap in hamster genome results in 114 amino acid truncation. Data is based on partial sequence

S1 Table. Homology search on Gpr103 from different species.
