# Supplementary material for: The hypothalamic RFamide, QRFP, increases feeding and locomotor activity: The role of Gpr103 and orexin receptors
Source: PLoS One. 2022 Oct 17;17(10):e0275604. doi: 10.1371/journal.pone.0275604 (PMC9576062; doi:10.1371/journal.pone.0275604)
Supplement: S2 Table — All comparisons are between male, homozygous wild-type and knock-out littermates. No differences in feeding parameters or in an oral glucose tolerance test were noted between genotypes. (PDF) [file pone.0275604.s011.pdf]

**Study**	**WT**	***Gpr103a* KO**	***Gpr103b* KO**
24 hour chow intake	4.17g ± 0.13 (n=14)	4.36g ± 0.32 (n=5)	4.51g ± 0.20 (n=6)
6-day cumulative chow intake	25.30g ± 0.77 (n=14)	26.78g ± 1.81 (n=5)	26.46g ± 1.71 (n=6)
Overnight fast-induced refeed (chow)	1 hour: 0.61g ± 0.04<br>2 hours: 0.89g ± 0.049<br>4 hours: 1.44g ± 0.08<br>(n=12)	1 hour: 0.57g ± 0.05<br>2 hours: 0.72g ± 0.05<br>4 hours: 1.25g ± 0.09<br>(n=4)	1 hour: 0.77g ± 0.09<br>2 hours: 1.02g ± 0.08<br>4 hours: 1.62g ± 0.09<br>(n=6)

S2 Table. Additional phenotypic data for Gpr103a and Gpr103b knock-out mice. All comparisons are between male, homozygous wild-type and knock-out littermates. No differences in feeding parameters were noted between genotypes.
