# Supplementary material for: The hypothalamic RFamide, QRFP, increases feeding and locomotor activity: The role of Gpr103 and orexin receptors
Source: PLoS One. 2022 Oct 17;17(10):e0275604. doi: 10.1371/journal.pone.0275604 (PMC9576062; doi:10.1371/journal.pone.0275604)
Supplement: S3 Table — (PDF) [file pone.0275604.s012.pdf]

Transgene	Primer	Sequence	Band size
**Gpr103a**	YS102W	CAT TGC CCA CTG TGT ATC G	WT: 566 bp
	YS102S	GGA GGC AGG AAG CAT GAA G	KO: 794 bp
	YSNEO	CAT AGC CGA ATA GCC TCT CC	
**Gpr103b**	4972-41	ACA TGG ATC AAC TGT TGG TAA GG	WT: 285 bp
	4973-44	GTT GCC AAA GGC TAG GAC AAC	KO: 378 bp
**Cre**	Control F	GGT CAG CCT AAT TAG CTC TGT	650 bp
	Control R	GAT CTC CAG CTC CTC CTC TGT C	
	Target F	GCC CTG GAA GGG ATT TTT GAA GCA	259 bp
	Target R	ATG GCT AAT CGC CAT CTT CCA GCA	

S3 Table. Primers used for genotyping.
